# TIA1 is a gender-specific disease modifier of a mild mouse model of spinal muscular atrophy

**DOI:** 10.1038/s41598-017-07468-2

**Published:** 2017-08-03

**Authors:** Matthew D. Howell, Eric W. Ottesen, Natalia N. Singh, Rachel L. Anderson, Joonbae Seo, Senthilkumar Sivanesan, Elizabeth M. Whitley, Ravindra N. Singh

**Affiliations:** 1 Department of Biomedical Sciences, Iowa State University, Ames, Iowa 50011 USA; 2 Division of Endocrinology, Cincinnati Children’s Hospital Medical Center, Cincinnati, OH 45229 USA; 3 Department of Veterinary Pathology, Iowa State University, Ames, IA 50011-1250 USA; 4 Pathogenesis, LLC, Gainesville, Florida 32614 USA

## Abstract

Spinal muscular atrophy (SMA) is caused by deletions or mutations of *Survival Motor Neuron 1* (*SMN1*) gene. The nearly identical *SMN2* cannot compensate for *SMN1* loss due to exon 7 skipping. The allele C (*C* ^+/+^) mouse recapitulates a mild SMA-like phenotype and offers an ideal system to monitor the role of disease-modifying factors over a long time. T-cell-restricted intracellular antigen 1 (TIA1) regulates *SMN* exon 7 splicing. *TIA1* is reported to be downregulated in obese patients, although it is not known if the effect is gender-specific. We show that female *Tia1-*knockout (*Tia1* ^−/−^) mice gain significant body weight (BW) during early postnatal development. We next examined the effect of *Tia1* deletion in novel *C* ^+/+^/*Tia1* ^−/−^ mice. Underscoring the opposing effects of *Tia1* deletion and low SMN level on BW gain, both *C* ^+/+^ and *C* ^+/+^/*Tia1* ^−/−^ females showed similar BW gain trajectory at all time points during our study. We observed early tail necrosis in *C* ^+/+^/*Tia1* ^−/−^ females but not in males. We show enhanced impairment of male reproductive organ development and exacerbation of the *C* ^+/+^/*Tia1* ^−/−^ testis transcriptome. Our findings implicate a protein factor as a gender-specific modifier of a mild mouse model of SMA.

## Introduction

Spinal muscular atrophy (SMA) is an autosomal recessive disease and a leading genetic cause of infant mortality^1, 2^. In >95% of cases, SMA is caused by the deletion, mutation and/or conversion of *survival motor neuron 1* (*SMN1*), a gene that codes for the SMN protein^3, 4^. Humans carry the paralog gene *SMN2* that does not compensate for the loss of *SMN1* due to predominant skipping of exon 7. Exon 7 skipping leads to production of SMNΔ7, an unstable and only partially functional protein^5–9^. Strategies aimed at the correction of *SMN2* exon 7 splicing hold the promise for SMA therapy^10^. SMN is a multifunctional protein with diverse roles in RNA metabolism, DNA repair and cell signaling^11^. SMA patients exhibit a spectrum of phenotypes from infants that present with symptoms at or soon after birth and die within two years (type I), infants that are able to sit up unaided but not ambulate and who can survive into teens or adulthood (type II), patients that are able to ambulate independently with normal or near-normal lifespan (type III) and patients that develop symptoms in adulthood (type IV)^2^. Aberrant expression and/or localization of SMN has also been associated with other pathological conditions including amyotrophic lateral sclerosis (ALS), inclusion body myositis and osteoarthritis^12–14^.

**Figure 1:**
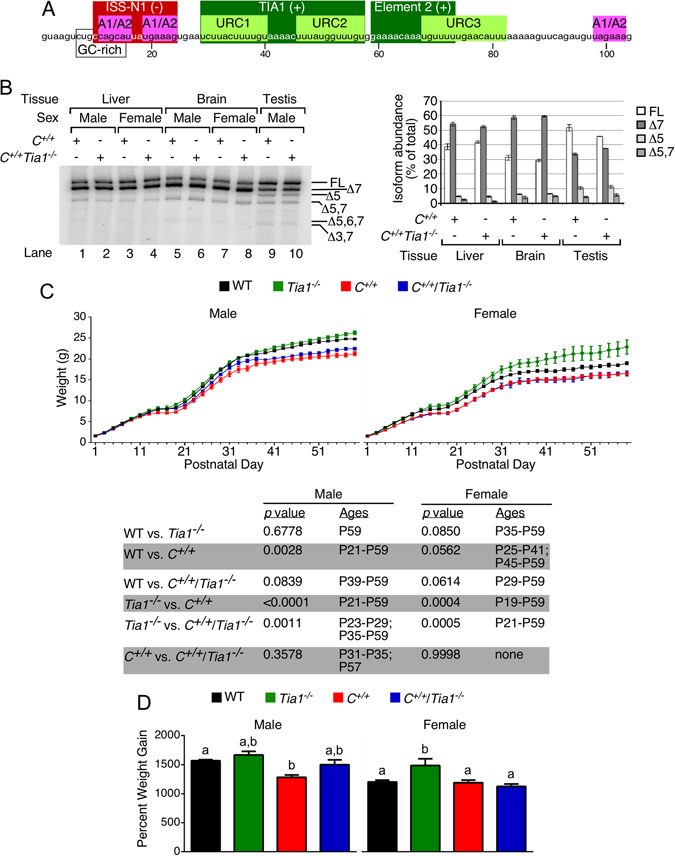
Gender-specific modulation of body weight with *Tia1* knockout. (**A**) Diagrammatic representation of the first 104 nucleotides of *SMN2* intron 7 (numbering begins from intron 7 beginning). Positive cis-elements (TIA1 and Element 2) that promote exon 7 inclusion and negative cis-element that promote exon 7 skipping (ISS-N1) are indicated by (+) and (−), respectively. hnRNPA1/A2 binding sites are also shown. Diagram adapted from^20^. (**B**) Representative semi-quantitative radioactive RT-PCR gel showing the results of MESDA^35^ to determine the abundance of *SMN2* splice variants. Tissue and mouse genotype are indicated above, lane numbers are indicated below and isoforms are indicated to the right of the gel, respectively. The graph presents the results of densitometry (n = 2 mice per tissue, sex and genotype). The intensity for each isoform band is expressed as a percent of the total intensity for each lane. Since there were no significant differences between values for male and female mice, values from both sexes were combined when calculating relative abundances of isoforms. (**C**) BW from P1 until P59 for male (left) and female (right) mice. For male mice, n = 5, 12, 6 and 7 mice for WT, *Tia1* ^−/−^, *C* ^+/+^ and *C* ^+/+^/*Tia1* ^−/−^ genotypes, respectively. For female mice, n = 5, 4, 6 and 5 mice for WT, *Tia1* ^−/−^, *C* ^+/+^ and *C* ^+/+^/*Tia1* ^−/−^ genotypes, respectively. Statistical comparison between the genotypes was performed with a repeated-measures two-way ANOVA followed by Tukey’s multiple comparison test. Details about these statistical comparisons appear below the graphs. For each sex, the *p* value indicates the result of Tukey’s multiple comparison test and the Ages column indicates at which ages the weight was significantly different between the specified genotypes. (**D**) Percent weight gain for male (left) and female (right) mice from P1 until P59 determined by dividing the P59 by the P1 weight. Errors bars on all graphs indicate S.E.M. For (D), statistical comparison between genotypes of the same sex was performed using a one-way ANOVA with Tukey’s multiple comparisons test and the results are denoted by lowercase letters. Bars with different letters indicate a statistically significant different between the means, whereas bars with the same letters are not significantly different (*p* < 0.05).

Multiple cis-elements and transacting factors have been implicated in regulation of *SMN* exon 7 splicing^15^. The first *in vivo* selection of an entire exon revealed a weak 5′ splice site (5′ss) as the limiting factor for exon 7 inclusion^16^. Consistently, several negative cis-elements are clustered around the 5′ss of *SMN* exon 7. These elements include the terminal stem-loop 2 (TSL2), intronic splicing silencer N1 (ISS-N1), a GC-rich sequence (GCRS) partially overlapping ISS-N1 and a RNA structure formed by a long-distance interaction (Fig. 1A)^17–20^. Negative cis-elements offer therapeutic targets for an antisense oligonucleotide (ASO)-mediated splicing correction in SMA^21^. Nusinersen, an ASO that targets ISS-N1, has been recently approved as the first therapy for SMA^22^. Uridine-rich clusters (URCs) immediately downstream of ISS-N1 serve as positive cis-elements and provide binding sites for the T-cell restricted intracellular antigen 1 (TIA1; Fig. 1A), a RNA binding protein with three RNA-recognition motifs (RRMs) and a glutamine-rich domain^23^. Consistently, a *TIA1* mutation that leads to the pathogenic condition called Welander distal myopathy (WDM) also negatively impacts splicing of *SMN* exon 7^24, 25^. However, it is not known if the pathogenicity of the mutated TIA1 protein is linked to the acquisition of a dominant negative property that adversely affects TIA1 functions. Of note, depletion of TIA1 and/or TIA1-related protein (TIAR) only partially affects splicing of exon 7 of endogenous *SMN*, suggesting that these proteins are dispensable for the regulation of *SMN* exon 7 splicing^23^. TIA1 and TIAR have a broad role in health and disease as they modulate inflammation, cellular stress, apoptosis and oncogenesis^26^. TIA1 is a key player in stress granule (SG) formation, an essential cellular process in which SMN also participates^27, 28^. Deletion of *Tia1* in mice causes wide-ranging pathological conditions from embryonic lethality to long-living animals with worsened pulmonary inflammation and dysregulated lipid homeostasis as well as abnormal membrane dynamics in the cerebellum and spinal cord^29^. However, it is not known if the loss of *Tia1* has an age and/or gender-specific effect on the phenotype of mice.

Recapitulating the broad pathophysiology in humans, several mouse models of SMA have been developed^30, 31^. The recently reported allele C (*C* ^+/+^) mouse model shows a mild phenotype without any adverse effect on lifespan^32^. *C* ^+/+^ mice carry a 42-kb human genomic sequence encompassing the *SMN2* gene at the locus adjacent to the mutated mouse *Smn* gene in which a portion of intron 6 through exon 8 is replaced with the equivalent sequence of human *SMN2* ^32^. The defining features of the *C* ^+/+^ model are reduced body weight (BW), peripheral necrosis and bradycardia^32^. Underscoring the high requirement of SMN in male reproductive organ development, *C* ^+/+^ mice show reduced testis size, degenerated seminiferous tubules, low sperm count and reduced male fertility^33^. We recently uncovered gender-specific amelioration of tail necrosis in *C* ^+/+^ mice when treated with an ASO that modestly increases the peripheral SMN levels due to correction of *SMN2* exon 7 splicing^34^. Our results also revealed that a modest peripheral increase in SMN levels substantially improved the reproductive phenotype of *C* ^+/+^ males^34^. These findings support the unique utility of the *C* ^+/+^ model in assessing the long-term effects of SMN-modulating compounds or factors on the gender-specific modification of disease severity.

Here we describe a novel *C* ^+/+^/*Tia1* ^−/−^ mouse model in which we have examined the effect of *Tia1* deletion on the background of the *C* ^+/+^ mouse model. We compare various phenotypical, histochemical and biochemical properties of *Tia1* ^−/−^, *C* ^+/+^ and *C* ^+/+^/*Tia1* ^−/−^ mice with wild type (WT) mice. We show that the deletion of *Tia1* itself has a gender- and age-specific effect on BW gain. Interestingly, the low level of SMN in *C* ^+/+^/*Tia1* ^−/−^ mice eliminates the female-specific BW gain characteristic to *Tia1* ^−/−^ mice. We observed an age- and gender-specific effect on tail necrosis in *C* ^+/+^/*Tia1* ^−/−^ mice. We also recorded gender-specific differences in the expression of a number of genes in brain of *C* ^+/+^/*Tia1* ^−/−^ mice. Further, *C* ^+/+^/*Tia1* ^−/−^ mice showed exacerbated developmental defects in male but not in female reproductive organs. Consistently, we recorded enhanced perturbations in expression of various genes in *C* ^+/+^/*Tia1* ^−/−^ testes compared to *C* ^+/+^ testes. Our findings bring a new perspective towards understanding of the role of the disease modifiers of SMA and other diseases that are likely to be impacted by aberrant expression of TIA1.

## Results

### Generation and partial characterization of the novel *C*^+/+^/*Tia1*^−/−^ mouse model

To examine the effect of TIA1 loss in mild SMA, we generated *C* ^+/+^/*Tia1* ^−/−^ mice. All mice used in our study were on a fully congenic C57Bl/6 background. Of note, the previously reported *Tia1* ^+/−^ mice were generated on the BALB/c background and when bred there was ~50% lethality of *Tia1* ^−/−^ pups between late embryonic development and three weeks of age^29^. To evaluate the penetrance of the embryonic lethality, we counted the number of *Tia1* ^−/−^ pups generated by our *Tia1* ^+/−^ breeding pairs. We observed no significant difference in the observed and the expected *Tia1* ^−/−^ genotype for the generated pups, suggesting that the congenic C57Bl/6 background does not lead to the lethality of *Tia1* ^−/−^ mice (Supplementary Table 1). We used *C* ^+/−^/*Tia1* ^−/−^ breeding pairs to generate *C* ^+/+^/*Tia1* ^−/−^ mice. Similar to *C* ^+/+^ and *Tia1* ^−/−^ pups, we observed no significant difference between the observed and the expected number of pups carrying *C* ^+/+^/*Tia1* ^−/−^ genotype (Supplementary Table 2). These results ruled out any potential lethal effect of the deletion of *Tia1* on the *C* ^+/+^ background.

To determine whether *Tia1* deletion affected *SMN2* splicing in *C* ^+/+^ mice, we used the multi-exon skipping detection assay (MESDA; Fig. 1B)^35^. In postnatal day 42 (P42) tissues, we did not observe a significant change in the abundance of any *SMN2* isoforms in the *C* ^+/+^/*Tia1* ^−/−^ as compared to *C* ^+/+^ brain and liver (Fig. 1B). These results were not totally surprising given the earlier observation that TIA1 depletion only partially affects *SMN* exon 7 splicing^23^. We observed a statistically insignificant increase in Δ7 transcripts in *C* ^+/+^/*Tia1* ^−/−^ testis compared to *C* ^+/+^ testis, suggesting that the recently reported postnatal switch of *SMN2* exon 7 splicing in the testis of young animals is not linked to TIA1 (Fig. 1B). We next monitored the BW of novel *C* ^+/+^/*Tia1* ^−/−^ mice from P1 until P59. In agreement with the previous report^32^, *C* ^+/+^ males were significantly lighter than WT males beginning at P27 (Fig. 1C). *Tia1* ^−/−^ males were slightly, but not significantly, heavier than WT males, especially during later time points, and significantly heavier than *C* ^+/+^ males beginning at P21 (Fig. 1C). The BW of the *C* ^+/+^/*Tia1* ^−/−^ males was generally greater than the BW of *C* ^+/+^ males, particularly so from P31 through P35 and at P57 (Fig. 1C). However, the *C* ^+/+^/*Tia1* ^−/−^ males were lighter than *Tia1* ^−/−^ and WT males (Fig. 1C). Beginning at P25, *C* ^+/+^ females were generally lighter than WT females (Fig. 1C). At P35 and later, *Tia1* ^−/−^ females were heavier than WT females (Fig. 1C). As opposed to males, there was no appreciable difference in BW at any time point for *C* ^+/+^ and *C* ^+/+^/*Tia1* ^−/−^ females (Fig. 1C). Further, *C* ^+/+^/*Tia1* ^−/−^ females were significantly lighter than WT and *Tia1* ^−/−^ females at around 3 to 4 weeks of age (Fig. 1C). When examining percent BW gain from P1 through P59, there was no notable difference between *C* ^+/+^/*Tia1* ^−/−^ males and all other genotypes, although *Tia1* ^−/−^ males gained significantly more BW than *C* ^+/+^ males (Fig. 1D). *C* ^+/+^/*Tia1* ^−/−^ females gained a similar percent of BW to females of all other genotypes, while *Tia1* ^−/−^ females gained significantly more BW than all other genotypes (Fig. 1D). Taken together, deletion of *Tia1* alone promoted a modest increase in BW compared to WT, especially in females, but when added to the *C* ^+/+^ background it only promoted BW gain in *C* ^+/+^/*Tia1* ^−/−^ males.

### Gender-specific progression of the peripheral necrosis in the *C*^+/+^/*Tia1*^−/−^ mouse model

**Figure 2:**
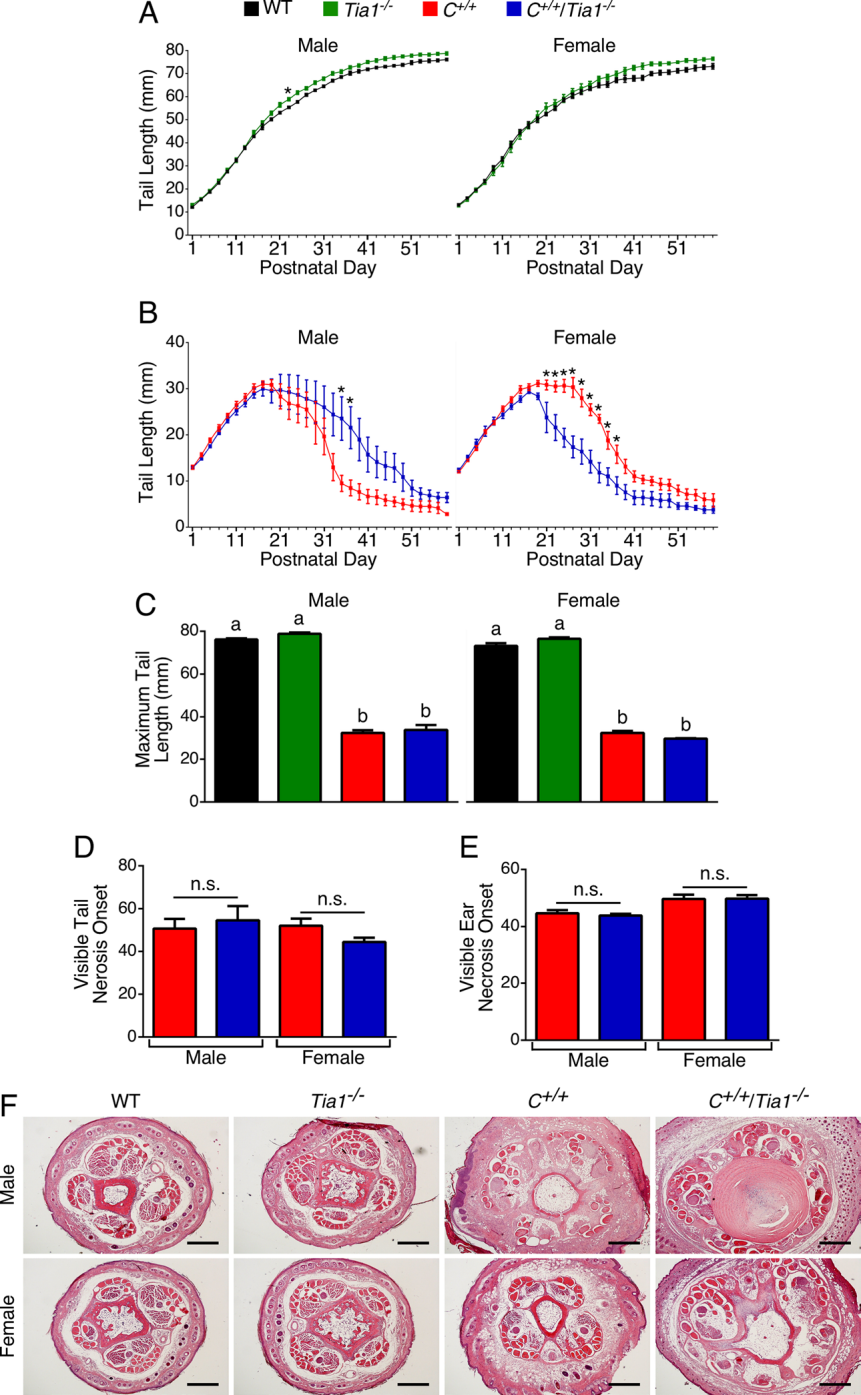
*Tia1* knockout alters tail necrosis progression in *C* ^+/+^ females. (**A**) Tail length from P1 until P59 for male (left) and female (right) WT and *Tia1* ^−/−^ mice. For males, n = 5 WT and n = 12 *Tia1* ^−/−^ mice. For females, n = 5 WT and n = 4 *Tia1* ^−/−^ mice. (**B**) Tail length from P1 until P59 for male (left) and female (right) *C* ^+/+^ and *C* ^+/+^/*Tia1* ^−/−^ mice. For males, n = 6 *C* ^+/+^ and n = 5 *C* ^+/+^/*Tia1* ^−/−^ mice. For females, n = 6 *C* ^+/+^ and n = 5 *C* ^+/+^/*Tia1* ^−/−^ mice. (**C**) Maximum observed tail length from P1 until P59 for male (left) and female (right) mice. (**D**) Age of visible tail necrosis onset for male and female *C* ^+/+^ and *C* ^+/+^/*Tia1* ^−/−^ mice. (**E**) Age of visible ear necrosis onset for male and female *C* ^+/+^ and *C* ^+/+^/*Tia1* ^−/−^ mice. Numbers of mice for (**C**,**D**) and (**E**) are the same as in (**A**) and (**B**). (**F**) Micrographs of tail base cross sections. Sex and genotype are indicated to the left and above the micrographs, respectively. Scale bar is 500 µm. Error bars on all graphs indicate S.E.M. For (**A**) and (**B**), for each sex, comparison between the genotypes was performed using a repeated measures two-way ANOVA with Tukey’s multiple comparison test. Statistical significance for (**A**) and (**B**) was denoted by an asterisk (*), indicating *p* < 0.05. For (**C**), comparison between the genotypes for each sex was performed using a one-way ANOVA with Tukey’s multiple comparison test. Statistical significance for (**C**) was denoted by lowercase letters; bars with different letters indicate a statistically significant difference between the means, whereas bars with the same letters are not significantly different (*p* < 0.05). For (**D** and **E**), comparison between the genotypes for each sex was performed using an unpaired two-tailed Student’s *t* tests. Abbreviation: n.s., not significant.

We next assessed the development and progression of tail necrosis in the *C* ^+/+^/*Tia1* ^−/−^mice. Deletion of *Tia1* alone resulted in a modest but significant increase in male tail growth (Fig. 2A; F(1,15) = 6.113; *p* = 0.0259). When combined with the *C* ^+/+^ background, *Tia1* knockout had no appreciable effect on the tail growth in males (Fig. 2B; F(1,11) = 2.079; *p* = 0.1772). For females, *Tia1* knockout alone did not impact tail growth (Fig. 2A, right panel; F(1,7) = 1.899; *p* = 0.2107). However, *Tia1* knockout in *C* ^+/+^ females did affect tail length (Fig. 2B, right panel; F(1,9) = 19.09; *p* = 0.0018). Female *C* ^+/+^/*Tia1* ^−/−^ tails were significantly shorter than *C* ^+/+^ tails from P21 until P37 (Fig. 2B). While *C* ^+/+^ and *C* ^+/+^/*Tia1* ^−/−^ maximum tail lengths were markedly reduced compared to WT and *Tia1* ^−/−^ mice, there was no difference in maximum tail length between *C* ^+/+^ and *C* ^+/+^/*Tia1* ^−/−^ mice for either sex (Fig. 2C). Although we noted an accelerated progression of necrosis in female *C* ^+/+^/*Tia1* ^−/−^ compared to *C* ^+/+^ tails (Fig. 2B), the age of visible tail necrosis onset was not significantly altered between the genotypes (Fig. 2D). There was also no difference in the visible tail necrosis onset in males (Fig. 2D). Further, we observed no appreciable difference in the onset of visible ear necrosis between *C* ^+/+^ and *C* ^+/+^/*Tia1* ^−/−^ mice of either sex (Fig. 2E). By P42, *C* ^+/+^ and *C* ^+/+^/*Tia1* ^−/−^ tails from both males and females exhibited similar pathology, including swelling, tissue disorganization and the beginning of vascular necrosis (Fig. 2F). Taken together, our results indicate that *Tia1* knockout on the *C* ^+/+^ background hastened tail loss, but did not advance the onset of necrosis.

### Severely impaired male reproductive organ development in the *C*^+/+^/*Tia1*^−/−^ mouse model

**Figure 3:**
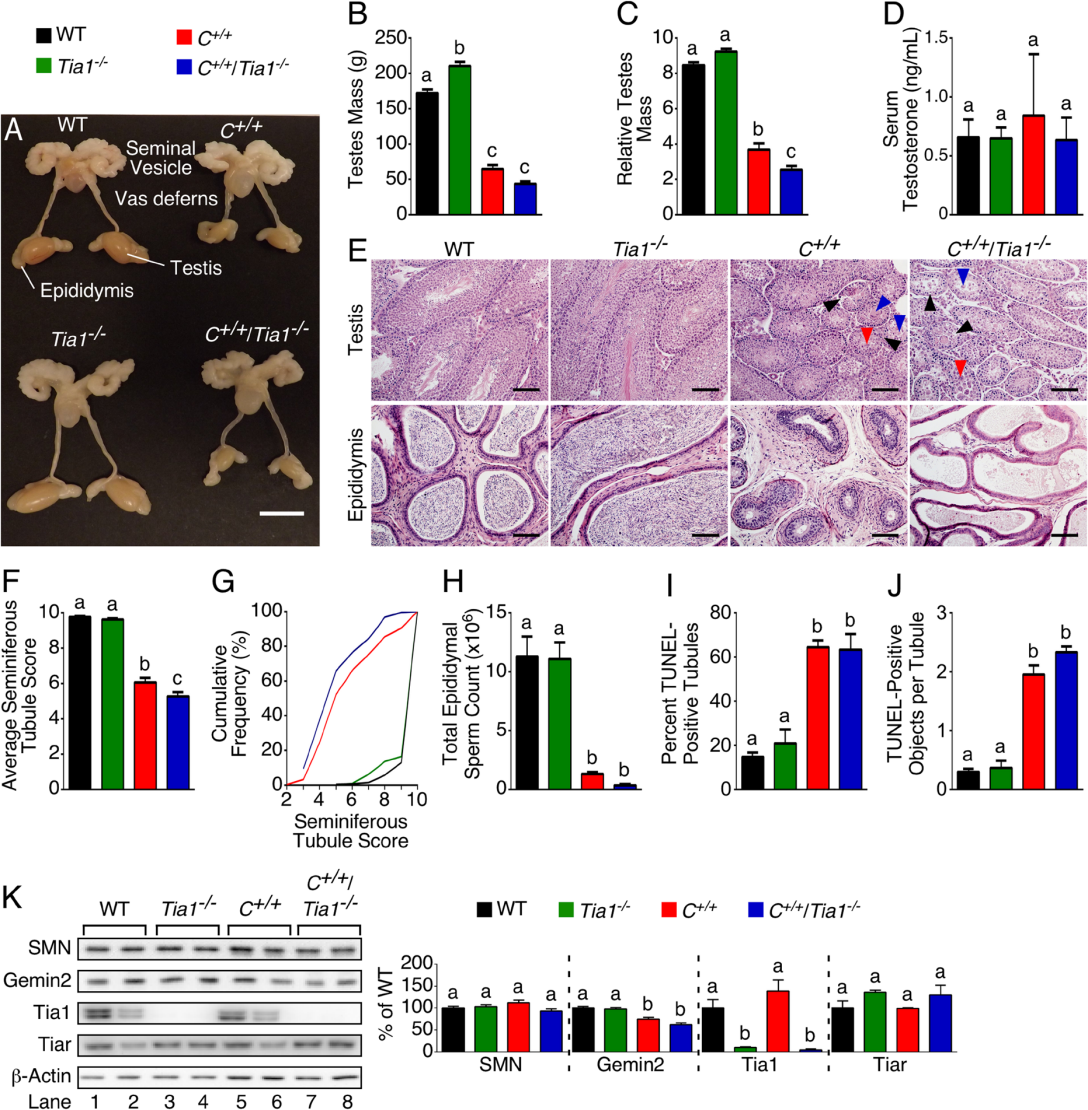
*Tia1* knockout exacerbates the P42 *C* ^+/+^ testis phenotype. (**A**) Male reproductive organs, including testis, epididymis, vas deferens and seminal vesicle, from P42 mice. Scale bar is 10 mm. (**B**) Gross testes mass for P42 mice (n = 10, 8, 8 and 7 WT, *Tia1* ^−/−^, *C* ^+/+^ and *C* ^+/+^/*Tia1* ^−/−^ mice, respectively). (**C**) Relative testes mass for P42 mice as determined by dividing the testis mass by total BW. Numbers of mice the same as in (**B**). (**D**) Serum testosterone level for P42 mice (n = 4 mice per genotype). (**E**) Representative H&E-stained cross sections from the testis and the cauda epididymis. Genotypes are indicated above each micrograph. Examples of vacuolization (black arrowheads), multinucleated bodies (red arrowheads) and sloughed tissue (blue arrowheads) are marked. Scale bar is 100 µm. (**F**) Seminiferous tubule health was assessed with a 10-point scale (see Materials and Methods). The graph indicates the average score for each genotype. Numbers of mice are the same as in (**B**). (**G**) Cumulative frequency plot of seminiferous tubule scores for all mice. (**H**) Total epididymal sperm count from P60 mice (n = 8, 12, 6 and 8 WT, *Tia1* ^−/−^, *C* ^+/+^ and *C* ^+/+^/*Tia1* ^−/−^ mice, respectively). (**I**) Percentage of seminiferous tubules with at least one TUNEL-positive object as assessed by TUNEL staining. A minimum of 50 seminiferous tubules per mouse were examined (n = 4 mice per genotype). (**J**) Average number of TUNEL-positive objects per seminiferous tubule (n = 4 mice per genotype). (K) Representative western blots for SMN, Gemin2, Tia1, Tiar and loading control β-actin in P42 testis. Full-length blots are presented in Supplementary Fig. 1. Genotypes and lane numbers are indicated above and below the blots, respectively. The graphs to the right indicate the results of densitometry (n = 4 mice for each genotype). Error bars on all graphs indicate S.E.M. For (**B**,**C**,**D**,**F**,**H**,**I** and **J**) and (**K**), statistical comparison between genotypes was performed using a one-way ANOVA with Tukey’s multiple comparison test and the results are denoted by lowercase letters. Bars with different letters indicate a statistically significant different between the means, whereas bars with the same letters are not significantly different (*p* < 0.05). For (**B**,**C**,**D**,**F**,**H**,**I** and **J**), data from WT and *C* ^+/+^ mice was previously published^33^.

We have recently shown that *C* ^+/+^ mice display impaired testicular development and function^33^. Therefore, we examined whether deletion of *Tia1* would worsen the reproductive phenotype of *C* ^+/+^ males. At P42, *C* ^+/+^/*Tia1* ^−/−^ testes were smaller than WT and *Tia1* ^−/−^ testes (Fig. 3A–C), and the relative mass of *C* ^+/+^/*Tia1* ^−/−^ testes was significantly reduced compared to *C* ^+/+^ testes (Fig. 3C). However, the serum level of testosterone, a hormone important for sexual maturation, was very similar for all genotypes under investigation (Fig. 3D). Both *C* ^+/+^/*Tia1* ^−/−^ and *C* ^+/+^ testes exhibited a heterogeneous mixture of seminiferous tubule morphology and evidence of tubular degeneration (Fig. 3E). We scored seminiferous tubule architecture on a previously described 1-to-10 scale in which 1 and 10 represent complete loss of cells and complete progression of spermatogenesis, respectively^33, 36^. The average score of seminiferous tubules from *C* ^+/+^/*Tia1* ^−/−^ testis was significantly reduced compared to *C* ^+/+^ testis (Fig. 3F). The overall graphical distribution of scores for *C* ^+/+^/*Tia1* ^−/−^ testes was shifted to the left (more severe pathology) relative to *C* ^+/+^ testes (Fig. 3G). This finding indicates that a greater proportion of the seminiferous tubules from *C* ^+/+^/*Tia1* ^−/−^ testes were affected more than in *C* ^+/+^ testes, with reduced numbers of post-meiotic cells, indicative of more severely impaired spermatogenesis. Consistently, spermatozoa were notably reduced in the cauda epididymis of *C* ^+/+^/*Tia1* ^−/−^ mice (Fig. 3E) and the total sperm count was less than 5% of the WT and *Tia1* ^−/−^ mice and only ~30% of the *C* ^+/+^ mice (Fig. 3H). As previously reported, the P42 *C* ^+/+^ testis exhibits prominent TUNEL staining, demonstrative of increased apoptosis^33^. While the percent of TUNEL-positive seminiferous tubules (Fig. 3I) and TUNEL-positive objects per tubule were significantly increased in *C* ^+/+^/*Tia1* ^−/−^ testes compared to WT and *Tia1* ^−/−^ testes, there was no difference in these measures compared to *C* ^+/+^ testis (Fig. 3I,J). To assess potential reasons for *Tia1* knockout exacerbation of the *C* ^+/+^ male reproductive phenotype, we examined the expression of SMN and its key interacting partner, Gemin2, in the P42 testis. We also examined the levels of Tiar, a close homolog of Tia1. While Gemin2 was significantly reduced in *C* ^+/+^/*Tia1* ^−/−^ compared to WT and *Tia1* ^−/−^, it was no different compared to *C* ^+/+^ testis (Fig. 3K). As expected, Tia1 protein was not detected in *Tia1* ^−/−^ and *C* ^+/+^/*Tia1* ^−/−^ testes. Also, we did not observe a compensatory upregulation of Tiar protein in *Tia1* ^−/−^ and *C* ^+/+^/*Tia1* ^−/−^ testes (Fig. 3K).

In contrast to males, *C* ^+/+^ female reproductive organs do not display any abnormalities^33^. We examined the uterus/ovary phenotypes and observed a trend for a decrease in uterus/ovary mass for *C* ^+/+^/*Tia1* ^−/−^ females compared to the other genotypes (Supplementary Fig. 2A–C). However, uterus/ovary histology appeared similar in all the genotypes (Supplementary Fig. 2D). SMN and Gemin2 proteins were significantly reduced in *C* ^+/+^/*Tia1* ^−/−^ uterus/ovary, but not significantly different compared to *C* ^+/+^ uterus/ovary (Supplementary Fig. 2E). As expected, Tia1 protein was absent in *Tia1* ^−/−^ uterus/ovary, and there was no compensatory upregulation of Tiar (Supplementary Figs 2E and 3).

### Histological phenotypes and protein expression levels in the peripheral tissues of the *C*^+/+^/*Tia1*^−/−^ mouse model

Recent reports indicate abnormal development of the intestine, liver and spleen in the severe Taiwanese type I mice^37–39^. These findings indicate that reduced SMN has an adverse effect on the development of peripheral tissues. To capture abnormalities in peripheral tissues in *C* ^+/+^/*Tia1* ^−/−^ mice, we performed histology on intestine, liver, spleen and heart. As controls, we also examined age-matched samples from WT, *Tia1* ^−/−^ and *C* ^+/+^ mice. We observed no histological evidence of abnormalities for any of these tissues in *Tia1* ^−/−^, *C* ^+/+^ or *C* ^+/+^/*Tia1* ^−/−^ males or females (data not shown). These results suggest that SMN protein level is not low enough in *C* ^+/+^/*Tia1* ^−/−^ mice to impair the development of intestine, liver, spleen and heart. We also examined whether deletion of *Tia1* would cause a gender-specific alteration in expression of SMN, Gemin2 and Tiar in brain, spinal cord and heart of *C* ^+/+^/*Tia1* ^−/−^ mice. While SMN and Gemin2 were markedly reduced in brain of both male and female *C* ^+/+^/*Tia1* ^−/−^ mice compared to the age-matched WT and *Tia1* ^−/−^ mice, the levels of these two proteins were comparable to that observed in *C* ^+/+^ brain (Supplementary Figs 4A and 5). Similarly, SMN and Gemin2 were markedly reduced in *C* ^+/+^/*Tia1* ^−/−^ spinal cord, but not different from the *C* ^+/+^ spinal cord (Supplementary Figs 4B and 6). In the heart, an organ shown to be abnormal in the severe Δ7 model^40–42^ as well as in *C* ^+/+^ mice^32^, SMN protein was significantly reduced in *C* ^+/+^/*Tia1* ^−/−^ compared to WT and *Tia1* ^−/−^, but not different from *C* ^+/+^ mice (Supplementary Figs 4C and 7). Interestingly, Gemin2 expression was similar in hearts of all four genotypes (Supplementary Fig. 4C). As expected, Tia1 protein was not detected in brain, spinal cord and heart of *Tia1* ^−/−^ mice. Also, we did not observe any change in the level of Tiar in any of the samples obtained from mice with deleted *Tia1* (Supplementary Fig. 4).

### Transcriptome-wide changes in the brain of the *C*^+/+^/*Tia1*^−/−^ mouse model

We have previously shown that transcriptomes of brain and liver of *C* ^+/+^ mice are minimally impacted compared to WT mice (Sequence Read Archive accession number SRP062636)^33^. In order to determine whether this is still the case when *Tia1* is deleted, we performed RNA-seq on brain and liver samples of *C* ^+/+^/*Tia1* ^−/−^ mice (Sequence Read Archive accession number SRP106484). As controls, we included *C* ^+/+^ and *Tia1* ^−/−^ mice (Supplementary Fig. 8; Supplementary Table 3). Deletion of *Tia1* had only a very mild effect on the overall transcriptomes of brain and liver (Supplementary Fig. 8), although we observed significant changes in expression of 8 genes in brain when compared to WT brain (Supplementary Table 4). *C* ^+/+^/*Tia1* ^−/−^ brain showed altered expression of 17 genes compared to WT brain (Supplementary Table 4). Of these, 4 genes (*Cntn4*, *Spr-ps1*, *Nat8f5* and *Gm15631*) overlap with the genes affected in *Tia1* ^−/−^ brain. In addition to comparing the transcriptomes of each genotype to the WT transcriptome, we also compared the *C* ^+/+^/*Tia1* ^−/−^ transcriptome to *C* ^+/+^ and *Tia1* ^−/−^ transcriptomes (Supplementary Fig. 8). Only one gene, coding for histone protein (Hist1h4h) was significantly altered in *C* ^+/+^/*Tia1* ^−/−^ compared to *Tia1* ^−/−^ brain (Supplementary Table 4), confirming that most of the changes in this genotype are due to the loss of *Tia1*. When compared to *C* ^+/+^ brain, 4 genes that were affected in *Tia1* ^−/−^ brain showed altered expression in *C* ^+/+^/*Tia1* ^−/−^ brain (Supplementary Table 4). Overall, these results suggest that the combined effect of the *Tia1* deletion and the low SMN level has a greater impact on the transcriptome than the effect of either *Tia1* deletion or low SMN alone.

**Figure 4:**
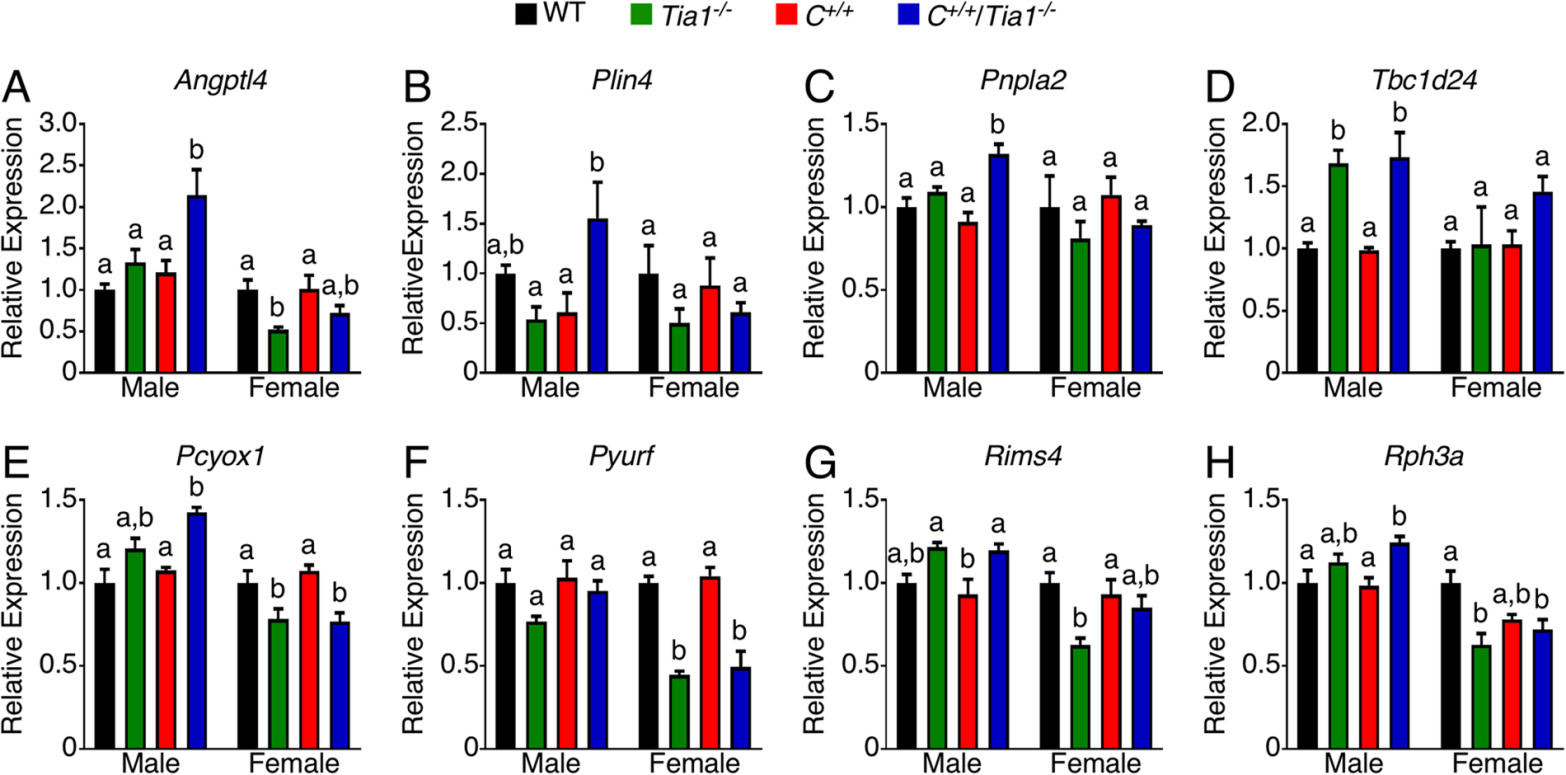
Gender-specific dysregulation of genes in P42 brain. (**A**–**H**) Expression of genes with gender-specific dysregulation in *Tia1* ^−/−^ and *C* ^+/+^/*Tia1* ^−/−^ brain. Expression is relative to WT of the same sex. For each gene, n = 4 mice per genotype and sex. Error bars on all graphs indicate S.E.M. For each gene, statistical comparison between the genotypes of the same sex was performed using a one-way ANOVA followed by Tukey’s multiple comparison test and the results are denoted by lowercase letters. Bars with different letters indicate a statistically significant different between the means, whereas bars with the same letters are not significantly different (*p* < 0.05).

QPCR analysis generally validated the gene expression changes captured by RNA-seq of the brain samples (Table 1). Three genes, *Cntn4*, *Herc6* and *Nat8f5*, were strongly downregulated in male and female *Tia1* ^−/−^ and *C* ^+/+^/*Tia1* ^−/−^ brain (Table 1). We noted *Tia1* deletion led to gender-specific changes in expression of several genes regardless of the *Smn* genotype (Fig. 4). The affected genes included *Angptl4*, *Plin4*, *Pnpla2* and *Tbc1d24*. These genes are involved in lipid storage and membrane trafficking and dynamics and were previously shown to be dysregulated in the *Tia1* ^−/−^ nervous system^43^, in addition to *Pcyox1*, *Pyurf*, *Rims4* and *Rph3a*, genes identified by our RNA-seq analysis (Fig. 4). When considering the *Smn* genotype, only *Acin1* was significantly different between female *Tia1* ^−/−^ (downregulated compared to WT and *C* ^+/+^) and female *C* ^+/+^/*Tia1* ^−/−^ (no different from WT and *C* ^+/+^) brain (Table 1). For male mice, expression of the lipid storage-related genes *Angptl4*, *Plin4* and *Pnpla2* were significantly upregulated in *C* ^+/+^/*Tia1* ^−/−^ compared to WT and *C* ^+/+^ brain (Fig. 4A–C). Notably, each of these genes was also dysregulated in a gender-specific manner.

**Table 1:** Select gene expression in female and male brain.

Gene	Female				Male			
	WT	*Tia1* ^−/−^	*C* ^+/+^	*C* ^+/+^/*Tia1* ^−/−^	WT	*Tia1* ^−/−^	*C* ^+/+^	*C* ^+/+^/*Tia1* ^−/−^
*Acin1*	1.00 ± 0.06^a,c^	0.64 ± 0.050^b^	1.01 ± 0.10^a,c^	0.92 ± 0.03^c^	1.00 ± 0.03^a,b^	1.05 ± 0.08^a^	0.83 ± 0.03^b^	1.05 ± 0.04^a^
*Cntn4*	1.00 ± 0.09^a^	0.50 ± 0.03^b^	1.02 ± 0.05^a^	0.60 ± 0.03^b^	1.00 ± 0.05^a^	0.77 ± 0.06^a,b^	0.90 ± 0.08^a,b^	0.70 ± 0.03^b^
*Gm15631*	1.00 ± 0.08^a^	0.37 ± 0.04^b^	1.07 ± 0.05^a^	0.55 ± 0.03^b^	1.00 ± 0.05^a^	0.83 ± 0.06^a^	1.07 ± 0.21^a^	0.65 ± 0.04^a^
*Herc6*	1.00 ± 0.03^a^	0.31 ± 0.03^b^	1.00 ± 0.08^a^	0.55 ± 0.11^b^	1.00 ± 0.12^a^	0.54 ± 0.07^b^	1.00 ± 0.08^a^	0.63 ± 0.11^a,b^
*Hist1h2bc*	1.00 ± 0.24^a^	1.02 ± 0.11^a^	0.90 ± 0.06^a^	1.33 ± 0.18^a^	1.00 ± 0.09^a^	1.24 ± 0.09^a^	0.99 ± 0.23^a^	1.34 ± 0.08^a^
*Hist1h4h*	1.00 ± 0.14^a,b^	0.74 ± 0.06^a^	1.45 ± 0.11^b^	1.17 ± 0.17^a,b^	1.00 ± 0.10^a^	1.01 ± 0.08^a^	1.64 ± 0.32^a^	1.59 ± 0.17^a^
*Lancl2*	1.00 ± 0.06^a^	0.58 ± 0.05^b,c^	0.88 ± 0.05^a,c^	0.70 ± 0.07^c^	1.00 ± 0.07^a^	1.08 ± 0.08^a^	0.97 ± 0.08^a^	1.10 ± 0.05^a^
*Mfsd2a*	1.00 ± 0.09^a^	0.64 ± 0.10^a^	0.82 ± 0.16^a^	0.73 ± 0.04^a^	1.00 ± 0.01^a^	0.80 ± 0.06^a^	0.84 ± 0.09^a^	1.09 ± 0.16^a^
*Nat8f5*	1.00 ± 0.07^a^	0.20 ± 0.01^b^	0.96 ± 0.15^a^	0.25 ± 0.01^b^	1.00 ± 0.09^a^	0.39 ± 0.03^b^	0.94 ± 0.16^a^	0.35 ± 0.05^b^
*Parva*	1.00 ± 0.11^a^	0.69 ± 0.07^b^	0.88 ± 0.05^a,b^	0.75 ± 0.03^a,b^	1.00 ± 0.06^a^	1.17 ± 0.10^a^	1.05 ± 0.15^a^	1.16 ± 0.04^a^
*Pisd-ps1*	1.00 ± 0.05^a^	0.89 ± 0.04^a^	1.39 ± 0.14^a^	1.28 ± 0.23^a^	1.00 ± 0.10^a,b^	1.23 ± 0.15^a,b^	0.79 ± 0.13^a^	1.49 ± 0.21^b^
*Pnpla7*	1.00 ± 0.03^a^	1.14 ± 0.08^a^	0.99 ± 0.10^a^	1.09 ± 0.09^a^	1.00 ± 0.04^a^	0.91 ± 0.11^a^	0.88 ± 0.11^a^	0.85 ± 0.08^a^
*Prrxl1*	1.00 ± 0.47^a^	0.61 ± 0.23^a^	0.73 ± 0.03^a^	0.54 ± 0.12^a^	1.00 ± 0.12^a^	0.62 ± 0.23^a^	0.86 ± 0.03^a^	0.65 ± 0.12^a^
*Rxrg*	1.00 ± 0.08^a^	0.82 ± 0.13^a^	1.12 ± 0.07^a^	0.94 ± 0.14^a^	1.00 ± 0.13^a^	1.32 ± 0.05^a^	1.23 ± 0.21^a^	1.56 ± 0.11^a^
*Six3*	1.00 ± 0.15^a^	0.69 ± 0.09^a^	1.01 ± 0.15^a^	1.10 ± 0.14^a^	1.00 ± 0.12^a^	1.23 ± 0.10^a,b^	1.40 ± 0.19^a,b^	1.72 ± 0.20^b^
*Spr-ps1*	1.00 ± 0.17^a^	1.77 ± 0.58^a^	1.13 ± 0.31^a^	2.13 ± 0.61^a^	1.00 ± 0.26^a^	1.20 ± 0.18^a^	1.16 ± 0.40^a^	1.64 ± 0.45^a^
*Tbc1d1*	1.00 ± 0.11^a^	1.02 ± 0.07^a^	0.89 ± 0.070^a^	1.11 ± 0.040^a^	1.00 ± 0.08^a^	0.79 ± 0.06^a^	0.96 ± 0.03^a^	1.02 ± 0.20^a^

For each sex, relative expression was determined using the ΔΔCt method. For each gene, the WT value was set at 1.00. Each value represents the mean relative expression ± S.E.M (n = 4 mice per sex and genotype). For each sex and gene, statistical comparison between the genotypes was performed using a one-way ANOVA followed by Tukey’s multiple comparison test and the results are denoted by superscript lowercase letters. Values with different superscript letters indicate a statistically significant difference between the means, whereas values with the same letter are not significantly different (*p* < 0.05).

### A highly perturbed transcriptome of the testis of the *C*^+/+^/*Tia1*^−/−^ mouse model

**Figure 5:**
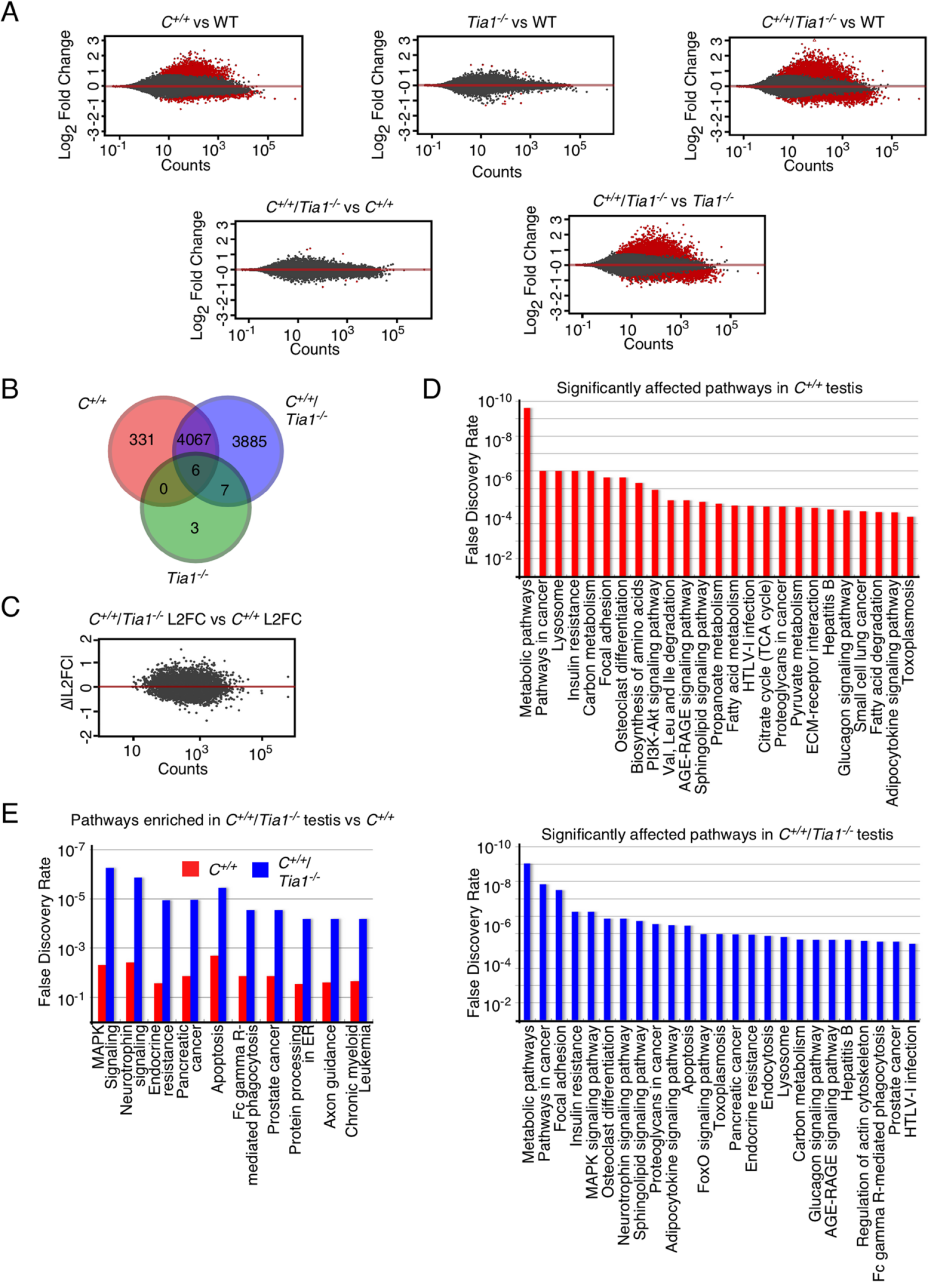
Knockout of *Tia1* in the *C* ^+/+^ background exacerbates perturbations in the testis transcriptome. (**A**) MA plots of estimated gene expression in testis for each mutant genotype compared to WT (top row) and *C* ^+/+^/*Tia1* ^−/−^ compared to each single mutant (bottom row). The y-axis depicts log_2_ fold change (L2FC) in gene expression in each comparison, and the x-axis depicts the mean read count for each gene between all samples. Each dot represents one gene, with red dots representing genes with significantly altered expression values (Benjamini and Hochberg (B+H) adjusted *p* value < 0.05). (**B**) Venn diagram of genes significantly altered in *C* ^+/+^, *Tia1* ^−/−^ and *C* ^+/+^/*Tia1* ^−/−^ testis compared to WT. Genotype is indicated next to each colored circle. (**C**) Modified MA plot depicting the difference in magnitude of expression changes between *C* ^+/+^ and *C* ^+/+^/*Tia1* ^−/−^ testes when compared to WT. ∆|L2FC| was obtained by taking the absolute value of L2FC for each genotype and subtracting the value obtained for *C* ^+/+^ testis from that of the value obtained for *C* ^+/+^/*Tia1* ^−/−^ testis. X-axis depicts the mean read count for each gene between all samples. Each dot represents one gene, all of which had significant expression changes in both mutant genotypes compared to WT. (**D**) KEGG pathways enriched for genes with altered expression levels in *C* ^+/+^ (top panel) and *C* ^+/+^/*Tia1* ^−/−^ (bottom panel) testes. The length of each bar represents the B+H false discovery rate of each pathway. (**E**) Ten KEGG pathways with the greatest change in enrichment between *C* ^+/+^ (red bars) and *C* ^+/+^/*Tia1* ^−/−^ (blue bars) testis. Length of bars represents the B+H false discovery rate of each pathway.

We have recently demonstrated that the low level of SMN during the early postnatal period in *C* ^+/+^ testis drastically changes the expression of thousands of genes affecting various pathways^33^. We performed RNA-seq to capture unique changes in the testis transcriptome as a consequence of the deletion of *Tia1* in *C* ^+/+^ mice. We observed that only 16 genes are significantly altered in *Tia1* ^−/−^ testis (Fig. 5A; Supplementary Table 5). However, *C* ^+/+^/*Tia1* ^−/−^ testis showed drastic changes in the transcriptome, as indicated by a wider spread of log_2_ fold change (L2FC) values than *C* ^+/+^ when each genotype was compared to WT (Fig. 5A; Supplementary Tables 6–9). We also noted a near doubling of genes with statistically significant changes in expression levels (7965 in *C* ^+/+^/*Tia1* ^−/−^ compared to 4404 in *C* ^+/+^). There was a significant (>90%) overlap of affected genes between *C* ^+/+^ and *C* ^+/+^/*Tia1* ^−/−^ testes (Fig. 5B). Also, we recorded substantial overlap (13 out of 16) of the aberrantly expressed genes between *Tia1* ^−/−^ and *C* ^+/+^/*Tia1* ^−/−^ testes. Interestingly, 6 genes (*Pde1c*, *Hpx*, *Fgg*, *Serpina1b*, *Serpina1e* and *Gm21847*) were altered in all 3 genotypes.

Results of QPCR validated the dysregulation of these genes except for *Gm21847* that was more strongly upregulated in *Tia1* ^−/−^ and *C* ^+/+^/*Tia1* ^−/−^ compared to *C* ^+/+^ testis (Table 2). Despite a substantial overlap of affected genes between *C* ^+/+^ and *C* ^+/+^/*Tia1* ^−/−^ testis, we observed 9 genes with significantly altered expression in *C* ^+/+^/*Tia1* ^−/−^ testis (Supplementary Table 5). *Atp6v1b1*, one of the affected testis genes in *C* ^+/+^/*Tia1* ^−/−^ mice was also affected in *Tia1* ^−/−^ relative to WT. In addition, *Snrnp27* was significantly upregulated in *Tia1* ^−/−^, but not in *C* ^+/+^ and *C* ^+/+^/*Tia1* ^−/−^ testis (Table 2).

**Table 2:** Select gene expression in testis.

Gene	WT	*Tia1* ^−/−^	*C* ^+/+^	*C* ^+/+^/*Tia1* ^−/−^
*1700124L16Rik*	1.00 ± 0.11^a^	1.43 ± 0.17^a^	1.17 ± 0.12^a^	0.89 ± 0.14^a^
*Aifm1*	1.00 ± 0.13^a^	1.17 ± 0.10^a,b^	1.44 ± 0.07^b^	1.44 ± 0.12^a,b^
*Akt1*	1.00 ± 0.18^a^	1.20 ± 0.09^a^	1.34 ± 0.13^a^	1.33 ± 0.11^a^
*Apaf1*	1.00 ± 0.19^a^	1.16 ± 0.18^a^	1.85 ± 0.25^a,b^	2.51 ± 0.28^b^
*Atp6v1b1*	1.00 ± 0.13^a^	0.14 ± 0.01^b^	0.77 ± 0.06^a^	0.06 ± 0.01^b^
*Bax*	1.00 ± 0.25^a^	1.00 ± 0.11^a^	1.99 ± 0.18^b^	2.42 ± 0.24^b^
*Capn2*	1.00 ± 0.07^a^	1.15 ± 0.11^a^	1.26 ± 0.12^a^	1.52 ± 0.16^a^
*Casp7*	1.00 ± 0.07^a^	1.08 ± 0.05^a^	1.50 ± 0.15^b^	1.83 ± 0.10^b^
*Casp9*	1.00 ± 0.25^a^	1.30 ± 0.13^a,b^	1.65 ± 0.13^b,c^	1.90 ± 0.07^c^
*Cpe*	1.00 ± 0.31^a^	1.04 ± 0.10^a^	2.36 ± 0.23^b^	2.59 ± 0.29^b^
*Cxcl12*	1.00 ± 0.21^a^	1.04 ± 0.05^a^	2.86 ± 0.28^b^	3.29 ± 0.17^b^
*Ddr1*	1.00 ± 0.19^a^	1.10 ± 0.06^a,b^	1.85 ± 0.12^b,c^	2.47 ± 0.48^c^
*Dhcr7*	1.00 ± 0.18^a^	1.10 ± 0.13^a^	1.23 ± 0.13^a^	1.38 ± 0.10^a^
*Dhcr24*	1.00 ± 0.20^a^	0.96 ± 0.09^a^	1.34 ± 0.17^a,b^	1.85 ± 0.10^b^
*Efnb1*	1.00 ± 0.04^a^	0.99 ± 0.11^a^	1.59 ± 0.15^b^	1.86 ± 0.14^b^
*Ephb1*	1.00 ± 0.24^a^	1.17 ± 0.17^a,c^	2.20 ± 0.24^b^	1.89 ± 0.13^b,c^
*Fgg*	1.00 ± 0.16^a^	0.71 ± 0.12^a,b^	0.46 ± 0.09^b^	0.51 ± 0.08^b^
*Gm9999*	1.00 ± 0.10^a^	0.90 ± 0.05^a^	0.63 ± 0.09^b^	0.36 ± 0.06^b^
*Gm21847*	1.00 ± 0.23^a^	6.98 ± 0.80^b^	2.03 ± 0.51^a^	4.29 ± 0.94^b^
*Herc6*	1.00 ± 0.20^a^	0.64 ± 0.11^a,b^	0.32 ± 0.09^b,c^	0.23 ± 0.02^c^
*Hpx*	1.00 ± 0.10^a^	0.24 ± 0.12^b^	0.28 ± 0.06^b^	0.27 ± 0.02^b^
*Itgb1*	1.00 ± 0.10^a^	1.04 ± 0.06^a^	1.43 ± 0.16^a,b^	1.84 ± 0.13^b^
*Lamb2*	1.00 ± 0.32^a^	1.01 ± 0.14^a^	2.06 ± 0.29^b^	2.63 ± 0.26^b^
*Lss*	1.00 ± 0.19^a^	1.23 ± 0.09^a,b^	1.73 ± 0.20^b^	1.90 ± 0.09^b^
*Msmo1*	1.00 ± 0.24^a^	0.99 ± 0.07^a^	2.36 ± 0.33^b^	2.34 ± 0.13^b^
*Nagk*	1.00 ± 0.28^a,b^	0.87 ± 0.07^a^	1.48 ± 0.12^b^	1.57 ± 0.08^b^
*Nat8f4*	1.00 ± 0.19^a^	0.77 ± 0.09^a,b^	0.48 ± 0.04^b^	0.47 ± 0.04^b^
*Neat1*	1.00 ± 0.07^a^	1.74 ± 0.18^a^	2.05 ± 0.29^b^	2.75 ± 0.41^b^
*Ntn1*	1.00 ± 0.17^a^	1.30 ± 0.04^a^	1.60 ± 0.13^b^	1.47 ± 0.11^a^
*Nup210*	1.00 ± 0.46^a^	0.65 ± 0.08^a^	0.45 ± 0.04^a^	0.59 ± 0.05^a^
*Oaz3*	1.00 ± 0.22^a^	1.04 ± 0.14^a^	0.88 ± 0.07^a,b^	0.51 ± 0.08^b^
*Pde1c*	1.00 ± 0.12^a^	0.73 ± 0.08^a,b^	0.65 ± 0.06^b^	0.64 ± 0.06^b^
*Ppp3r2*	1.00 ± 0.12^a^	1.16 ± 0.15^a^	0.93 ± 0.08^a^	0.77 ± 0.07^a^
*Robo1*	1.00 ± 0.17^a^	1.26 ± 0.10^a,b^	1.68 ± 0.11^b^	1.96 ± 0.11^b^
*RP23-402F16*	1.00 ± 0.22^a^	0.76 ± 0.05^a,b^	0.69 ± 0.06^a,b^	0.40 ± 0.07^b^
*Sema7a*	1.00 ± 0.37^a,c^	1.00 ± 0.11^a^	1.78 ± 0.17^b^	1.76 ± 0.05^b,c^
*Serpina1b*	1.00 ± 0.13^a^	0.62 ± 0.04^b^	0.67 ± 0.08^b^	0.58 ± 0.12^b^
*Serpina1e*	1.00 ± 0.17^a^	0.69 ± 0.06^a^	0.68 ± 0.06^a^	0.60 ± 0.13^a^
*Shank2*	1.00 ± 0.29^a^	0.79 ± 0.26^a^	0.87 ± 0.17^a^	0.64 ± 0.23^a^
*Snrnp27*	1.00 ± 0.24^a^	1.92 ± 0.20^b^	1.24 ± 0.09^a^	1.34 ± 0.10^a,b^
*Speer4cos*	1.00 ± 0.08^a^	2.81 ± 0.15^b^	1.07 ± 0.08^a^	2.25 ± 0.39^b^
*Srgap1*	1.00 ± 0.18^a^	1.10 ± 0.13^a^	1.23 ± 0.13^a^	1.38 ± 0.10^a^
*Unc5b*	1.00 ± 0.07^a^	1.06 ± 0.05^a^	1.13 ± 0.07^a^	1.04 ± 0.08^a^

Relative expression was determined using the ΔΔCt method. For each gene, the WT value was set at 1.00. Each value represents the mean relative expression ± S.E.M (n = 4, 6, 6 and 5 WT, *Tia1* ^−/−^, *C* ^+/+^ and *C* ^+/+^/*Tia1* ^−/−^ mice, respectively. For each gene, statistical comparison between the genotypes was performed using a one-way ANOVA followed by Tukey’s multiple comparison test and the results are denoted by superscript lowercase letters. Values with different superscript letters indicate a statistically significant difference between the means, whereas values with the same letter are not significantly different (*p* < 0.05).

The increased number of altered genes in *C* ^+/+^/*Tia1* ^−/−^ testis and the greater range of L2FC values compared to *C* ^+/+^ testis suggest that an additive effect of *Tia1* deletion and low level of SMN produces the radical changes in *C* ^+/+^/*Tia1* ^−/−^ testis transcriptome. In order to confirm this hypothesis, we extracted the L2FC values of the genes with altered expression in both genotypes. For each gene, we then subtracted the absolute value of the *C* ^+/+^ L2FC from the absolute value of *C* ^+/+^/*Tia1* ^−/−^ L2FC. This allowed us to directly compare the magnitude of expression deviation from WT. The values roughly clustered around 0 (Fig. 5C), confirming once again that the transcriptomes of the two genotypes are quite similar. However, when taken in aggregate, the overall trend was for a larger expression change in *C* ^+/+^/*Tia1* ^−/−^ testis (average ∆|L2FC| = 0.072). Consistently, there were 2323 genes with larger changes in *C* ^+/+^/*Tia1* ^−/−^ testis compared to 1750 in *C* ^+/+^ testis. The combined effect of *Tia1* deletion and low SMN levels on the expression of a subset of genes was even more apparent when we focused on genes with >0.5 difference in L2FC. In this case, 437 genes underwent larger expression changes in *C* ^+/+^/*Tia1* ^−/−^ compared to only 126 in *C* ^+/+^ testis.

In order to assign functional significance to the perturbed transcriptome of *C* ^+/+^/*Tia1* ^−/−^ testis, we tested the lists of genes with altered expression for enrichment of individual Kyoto Encyclopedia of Genes and Genomes (KEGG) pathways. As expected, there was a large amount of overlap between the two genotypes (Fig. 5D). However, the lists were not identical. For example, the lysosome and carbon metabolism pathways dropped from the third and the fifth most enriched pathways to #17 and #18 in the list, respectively. Conversely, several pathways, such as apoptosis, neurotrophin signaling, and endocytosis pathways are notably more strongly enriched in *C* ^+/+^/*Tia1* ^−/−^ testis compared to *C* ^+/+^ alone. Due to the enhanced phenotype of the *C* ^+/+^/*Tia1* ^−/−^ testis, we hypothesized that the key information towards determining the phenotype severity may be held within the pathways that are particularly enriched in *C* ^+/+^/*Tia1* ^−/−^ testis compared to *C* ^+/+^. In order to determine whether there was a potential bias towards certain pathways, we compared the false discovery rates (FDR) of all of the pathways enriched in both genotypes and extracted the 10 pathways with the largest changes in FDR (Fig. 5E). We observed a large number of signaling pathways, including mitogen-activated protein kinase (MAPK) and neurotrophin signaling, as well as axon guidance signaling that we previously characterized in *C* ^+/+^ testis^33^. We also noted a number of pathways involved in various cancers, and perhaps most telling, a strong increased enrichment of the apoptosis pathway.

### Radical changes in the expression of a subset of testicular genes of the *C*^+/+^/*Tia1*^+/+^ mouse model

**Figure 6:**
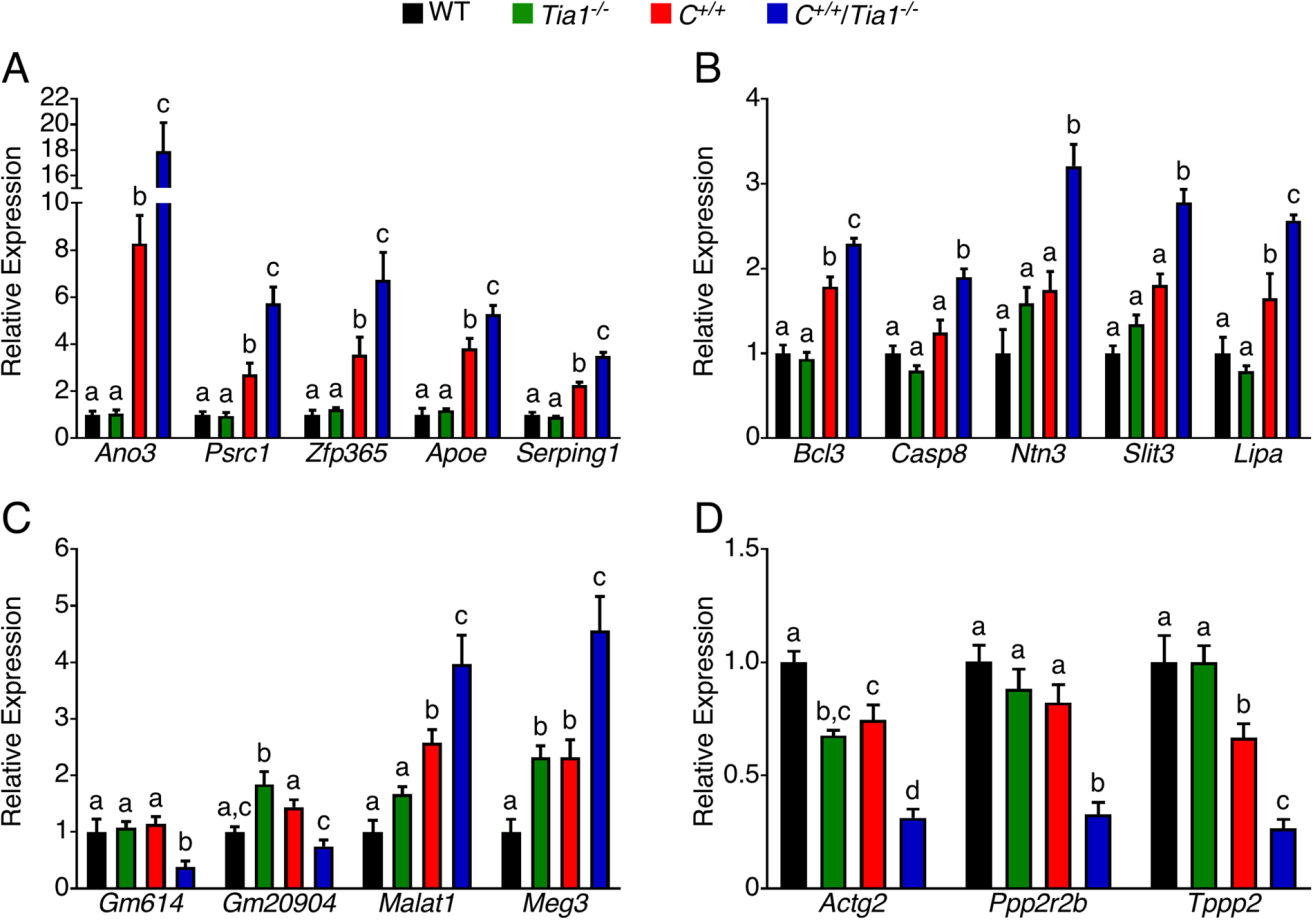
Genes significantly dysregulated in P42 *C* ^+/+^/*Tia1* ^−/−^ compared to *C* ^+/+^ testis. (**A**) Genes significantly upregulated in *C* ^+/+^/*Tia1* ^−/−^ compared to *C* ^+/+^ testis, including genes regulated by p53 (*Ano3*, *Psrc1* and *Zfp365*) and genes previously reported to be strongly upregulated in P42 testis (*Apoe* and *Serping1*)^33^. Expression is relative to WT. (**B**) Genes significantly upregulated in *C* ^+/+^/*Tia1* ^−/−^ compared to *C* ^+/+^ testis, including genes related to apoptosis (*Bcl3* and *Casp8*), axon guidance (*Ntn3* and *Slit3*) and steroid biosynthesis (*Lipa*). Expression is relative to WT. (**C**) lncRNA significantly dysregulated in *C* ^+/+^/*Tia1* ^−/−^ compared to *C* ^+/+^ testis. Expression is relative to WT. (**D**) Genes significantly downregulated in *C* ^+/+^/*Tia1* ^−/−^ compared to *C* ^+/+^ testis. Expression is relative to WT. For each gene, n = 4, 6, 6 and 5 WT, *Tia1* ^−/−^, *C* ^+/+^ and *C* ^+/+^/*Tia1* ^−/−^ mice, respectively. Error bars on all graphs indicate S.E.M. For each gene, statistical comparison between the genotypes was performed using a one-way ANOVA with Tukey’s multiple comparison test and the results are presented by lowercase letters. Bars with different letters indicate a statistically significant different between the means, whereas bars with the same letters are not significantly different (*p* < 0.05).

The expression of a subset of genes that were minimally impacted by the deletion of *Tia1* was drastically changed in *C* ^+/+^/*Tia1* ^−/−^ testes. We validated by QPCR the expression of several of these genes including *Ano3*, *Psrc1*, *Zfp365*, *Apoe*, *Serping1*, *Bcl3*, *Casp8*, *Ntn3*, *Slit3* and *Lipa* (Fig. 6A,B). Interestingly, expression of *Ano3* that codes for the K^+^ channel-regulating protein Anoactamin 3, was ~18-fold upregulated in *C* ^+/+^/*Tia1* ^−/−^ testes compared to ~8-fold upregulation in *C* ^+/+^ testis and no change in *Tia1* ^−/−^ testes (Fig. 6A). A significant increase in the anti-apoptotic factor *Bcl3* and the initiator caspase *Casp8* (Fig. 6B) correlated with a slight increase in TUNEL-positive cells indicative of apoptosis in *C* ^+/+^/*Tia1* ^−/−^ compared to *C* ^+/+^ testis (Fig. 3J). Consistently, the apoptosis pathway was significantly enriched in *C* ^+/+^/*Tia1* ^−/−^ compared to *C* ^+/+^ testis (Fig. 5E). Expression of a number of long non-coding RNAs (lncRNAs) including *Gm614*, *Gm20904*, *Malat1* and *Meg3* were significantly impacted in *C* ^+/+^/*Tia1* ^−/−^ testes compared to *C* ^+/+^ or *Tia1* ^−/−^ testes (Fig. 6C). In particular, the abundantly expressed *Malat1* and *Meg3* were >4-fold upregulated in *C* ^+/+^/*Tia1* ^−/−^ testes compared to <3-fold upregulation *C* ^+/+^ testis. Two lncRNAs, *Gm614* and *Gm20904*, were similarly downregulated specifically in *C* ^+/+^/*Tia1* ^−/−^ testes but not in *C* ^+/+^ or *Tia1* ^−/−^ testes compared to the WT testis. We also validated by QPCR the expression of a number of genes that were downregulated strongly in *C* ^+/+^/*Tia1* ^−/−^ testes but only weakly or not at all in *C* ^+/+^ or *Tia1* ^−/−^ testes compared to the WT testis. These included genes related to the cytoskeleton (*Actg2* and *Tppp2*) and a regulatory subunit for protein phosphatase 2 (*Ppp2r2b*) (Fig. 6D).

## Discussion

Here we report that the loss of TIA1 elicits a gender-specific effect on the progression of SMA-like symptoms in a mild SMA mouse model. While there are a few reported SMA disease modifiers, only Plastin3 has thus far been shown to be gender specific^44^. Oprea and colleagues demonstrated that female siblings that expressed high levels of the actin-binding protein Plastin3 displayed only a mild SMA phenotype despite carrying just two *SMN2* copies along with the absence of *SMN1* ^44^. Both TIA1 and SMN are multifunctional proteins involved in transcription, pre-mRNA splicing, mRNA stability/trafficking, SG formation, translation and apoptosis^11, 27, 28^. Due to the similar protein structure between TIA1 and TIAR, most of the TIA1-associated functions are expected to also be carried out by TIAR. However, as evidenced by a recently described *TIA1* mutation that leads to the human pathological condition WDM, TIA1 functions cannot be entirely replaced by TIAR. Consistent with the role of *TIA1* on *SMN* exon 7 splicing, WDM patients show increased skipping of *SMN* exon 7^24^. Inspired by these findings, we sought to determine the disease modifying role of TIA1 in SMA by employing the *C* ^+/+^ model that displays a mild SMA-like phenotype characterized by reduced BW and peripheral (tail and ear) necrosis. Additional hallmarks of mild SMA recapitulated in the *C* ^+/+^ mouse are defective male reproductive organ development, perturbed testicular transcriptome and male infertility^33^. A recent study demonstrated that the genetic background of a mouse has a modifying effect on the SMA disease phenotype, since an intermediate SMA mouse model generated on C57Bl/6 background produced a less severe phenotype than the same model generated on the FVB/NJ background^45^. This observation is not unique to SMA, since *Tia1* ^−/−^ mice employed in our study also show background-specific disease penetrance^29^. Unlike the ~50% lethality for *Tia1* ^−/−^ mice generated on BALB/c background, we recorded normal litter sizes for *Tia1* ^−/−^ mice generated on C57Bl/6 background. The reasons for the variable penetrance in the case of *Tia1* ^−/−^ mice generated on the BALB/c background remain unknown. We chose the C57Bl/6 background for our study due to the expected mild and homogenous phenotype that could be monitored over a longer period of time. Similar to *Tia1* ^−/−^ mice, we did not observe any lethality of *C* ^+/+^/*Tia1* ^−/−^ mice. Consistent with these findings, splicing of *SMN2* exon 7 was not significantly affected in most tissues of *C* ^+/+^/*Tia1* ^−/−^ mice. These results ruled out TIA1 as an essential regulator of *SMN2* exon 7 splicing and suggest that functions unrelated to *SMN2* exon 7 splicing contributed towards the distinct phenotype of *C* ^+/+^/*Tia1* ^−/−^ mice.

We performed this study keeping in mind that gender-specific differences may arise at any point during the disease progression. Indeed, we captured a significant BW gain in adult *Tia1* ^−/−^ females compared to the age-matched WT females, but no such change was observed in male mice (Fig. 1). Given the findings of an earlier study showing *TIA1* as one of the top-ranking genes to be downregulated in obese patients^46^, our results were not completely surprising. However, since the groups were not optimally balanced for age and gender (the majority of study subjects were females)^46^, specific inferences with respect to age and gender could not be drawn. Our results suggest that the downregulation of *TIA1* expression may be a better marker of obesity in females rather than in males. Surprisingly, our RNA-seq analysis did not reveal gene expression changes in the liver. However, downregulation of several genes in female *Tia1* ^−/−^ brain (Fig. 4) may contribute to BW gain, since the nervous system partly controls BW^47^. *Angptl4*, the source of Angiopoietin-like 4, is expressed throughout the brain, including the hypothalamus^48^, and regulates triglyceride metabolism and adiposity^49^. Knockout of *Angptl4* in mice leads to an obese phenotype and reduces energy expenditure; these changes are mediated through modulation of hypothalamic AMPK phosphorylation, which is a crucial cellular energy sensor^49^. Downregulation of *Angptl4* in female *Tia1* ^−/−^ brain could perturb the hypothalamic signaling that regulates food intake and BW. *Pcyox1* encodes Prenylcysteine Oxidase 1, an enzyme that catabolizes prenylated proteins and generates hydrogen peroxide (H_2_O_2_). PCYOX1 may be involved with lipid homeostasis since several studies have demonstrated that PCYOX1 is associated with circulating plasma LDL and VLDL in human patients^50–52^. Although the exact function of this gene in the brain is not known, reduced *Pcyox1* expression and enzymatic activity could reduce H_2_O_2_ production in brain regions including the hypothalamus. Since H_2_O_2_ participates in hypothalamic signaling, its reduction could disrupt signaling that regulates BW^53^. *Rph3a* encodes Rabphilin 3a, a protein involved in neurotransmitter release and stabilization of NMDA receptors in the hippocampus^54^. *Rims4* encodes a protein that also functions in neurotransmitter release. Downregulation of these exocytosis-related genes could indicate impairment with synaptic transmission. While the above genes are downregulated similarly in female *Tia1* ^−/−^ and *C* ^+/+^/*Tia1* ^−/−^ brain, we only observed a difference in BW between *Tia1* ^−/−^ and WT and not between *C* ^+/+^/*Tia1* ^−/−^ and *C* ^+/+^ mice. This finding suggests that the low SMN level could counter the effect of *Tia1* deletion on the BW gain in females. Future studies that follow *C* ^+/+^/*Tia1* ^−/−^ mice over a longer period of time will allow for assessment of the long-term consequences of reduced SMN coupled with Tia1 loss on BW and adiposity.

Tail necrosis is the most prominent visible phenotype of the *C* ^+/+^ mouse and it is likely mediated through impaired autonomic nervous system input and function in the tail vasculature^32^. Our tail necrosis results suggest that Tia1 can modulate a major SMA-like phenotype in a gender-specific manner. Female *C* ^+/+^/*Tia1* ^−/−^ tails were significantly shorter compared to *C* ^+/+^ females from P21 to P37, but there was no change in the age of initial necrosis onset (Fig. 2). Comparatively, there was no difference in male *C* ^+/+^ and *C* ^+/+^/*Tia1* ^−/−^ tail length at any time. This finding suggests that the loss of Tia1 specifically in the *C* ^+/+^ female may modulate autonomic nervous system input to negatively affect the tail vasculature, perhaps through reduced blood flow and/or accelerated blood vessel degeneration. While this modulation likely occurs at the tail vasculature and its nerve inputs, gene expression changes in female *C* ^+/+^/*Tia1* ^−/−^ brain could also contribute to the differential necrosis phenotype. For example, Angiopoietin-like 4 encoded by *Angptl4* is involved in angiogenesis and blood vessel development in the brain^55^. In addition, this protein protects the cerebrovasculature after ischemic stroke^56^. *Angptl4* downregulation in *C* ^+/+^/*Tia1* ^−/−^ brain could indicate problems with brain vasculature that impair normal neuronal and/or glial function. Abnormal angiogenesis and/or maintenance of the vasculature would correlate with a previous report of impaired capillary development in SMA patient muscle as well as the skeletal muscle and spinal cord in two severe mouse models of SMA^57^. Additionally, reduction of *Rph3a* and *Rims4* that are involved with neurotransmitter release could be demonstrative of abnormal synaptic transmission. Collectively, disruption of these functions could worsen autonomic control of tail blood vessels and contribute to the reduced tail length in *C* ^+/+^/*Tia1* ^−/−^ females. While *Angptl4*, *Rph3a* and *Rims4* were also downregulated in the brain of *Tia1* ^−/−^ females, the reduction of SMN combined with the Tia1 loss appeared to be required for the accelerated tail necrosis observed in *C* ^+/+^/*Tia1* ^−/−^ females. The gender-specific gene expression changes were the likely cause why we do not observe shorter tails in *C* ^+/+^/*Tia1* ^−/−^ males compared to *C* ^+/+^ males. It is also possible that sex hormones contributed to the gender-specific effect on tail necrosis.

Male mice exhibit distinct brain gene expression changes compared to females (Table 1, Fig. 4). Specifically, three lipid homeostasis-related genes, *Angptl4*, *Plin4* and *Pnpla2*, previously shown to be upregulated in *Tia1* ^−/−^ nervous tissue^43^, were also upregulated in *C* ^+/+^/*Tia1* ^−/−^ compared to *Tia1* ^−/−^ brain. *Plin4* (Perilipin 4) coats nascent lipid droplets in adipocytes to prevent them from lipase degradation^58^ and polymorphisms in this gene may be associated with obesity risk^59^. *Pnpla2* encodes a protein involved in fatty acid catabolism. The collective brain-specific upregulation of *Angptl4*, *Plin4* and *Pnpla2* may indicate that male *C* ^+/+^/*Tia1* ^−/−^ mice are undergoing through stress-like fasting conditions even when food is not restricted^43^. The combination of SMN reduction and Tia1 loss appears to induce this fasting-like stress specifically in males possibly due to gender-specific hormonal control.

We observed that SMN protein level was no different in P42 *C* ^+/+^/*Tia1* ^−/−^ compared to WT, *Tia1* ^−/−^ and *C* ^+/+^ testis (Fig. 3K). The absence of testis-specific SMN reduction in *C* ^+/+^ and *C* ^+/+^/*Tia1* ^−/−^ males is due to a *SMN2* splicing switch during the first wave of spermatogenesis that increases the relative amount of full-length transcripts^33^. Nevertheless, testis was one of the most affected tissues in *C* ^+/+^/*Tia1* ^−/−^ mice, likely due to sensitivity to SMN levels early in development before the splicing switch, as early postnatal treatment with a therapeutic ASO greatly ameliorates the testis phenotype^34^. In particular, the mitogen-activated protein kinase (MAPK) signaling pathway appeared to be drastically affected (Fig. 5E). MAPKs are stress-responsive signaling proteins linked to cellular survival and adaptation to stress^60^. In the testis, MAPK signaling plays a critical role in germ cell development and acrosome function, as well as in promoting germ cell apoptosis in response to stress conditions such as hyperthermia and hypoxia^61^. Tia1 is predicted to inhibit MAPK-induced apoptosis through sequestration of RACK1 in stress granules^62^. Consistently, apoptosis-linked pathways are also disproportionately affected in *C* ^+/+^/*Tia1* ^−/−^ testes compared to *C* ^+/+^ testes (Fig. 5E). For instance, two apoptosis-related genes, *Bcl3* and *Casp8*, were further upregulated in C^+/+^/*Tia1* ^−/−^ testis (Fig. 6B). Although there was no difference at P42 in TUNEL-positive cells per tubule between *C* ^+/+^ and *C* ^+/+^/*Tia1* ^−/−^ genotypes (Fig. 3J), it is possible that enhanced apoptosis occurs at an earlier age and contributes to the further deterioration of seminiferous tubules. Upregulation of several axon guidance-related genes, including *Ntn1*, *Ntn3*, *Slit3* and *Robo1* (Table 2) may also be related to dysregulated apoptosis. While ROBO and SLIT prevent axons from migrating to inappropriate locations during nervous system development, they have also been implicated in development of the ovary^63–65^. Ntn1 (Netrin1) can bind to Deleted in Colorectal Cancer (DCC) to promote DCC multimerization to provide pro-survival signals^66^. Loss of Ntn1 and DCC interaction can lead to caspase activation and apoptosis^64^. While it is not known if this pathway is active in the testis, *Ntn3* and *Slit3* are both significantly upregulated in C^+/+^/*Tia1* ^−/−^ testis (Fig. 6B) and could interfere with this regulation. For example, increased Slit3 could sequester Ntn1 and prevent its binding to DCC. Additionally, Ntn3 can also bind to DCC, albeit with lower affinity than Netrin1^67^. Binding of Ntn3 to DCC could disrupt DCC multimerization, reduce pro-survival signals and lead to caspase activation and apoptosis.

Pathways associated with cancer are also enriched in the *C* ^+/+^/*Tia1* ^−/−^ testis, and the genes *Psrc1*, *Ano3* and *Zfp365*, all upregulated in *C* ^+/+^/*Tia1* ^−/−^ testis (Fig. 6A), are regulated by the tumor suppressor p53^68–70^. PSRC1, also known as DDA3, encodes a microtubule-regulating protein that interacts with KIF2a to control chromosome alignment during metaphase and the spindle apparatus^71^. Further, human DDA3 is an oncoprotein that is downregulated by p53^72^. *Zfp365* encodes a zinc finger protein that can be directly upregulated by p53 to suppress fragile sites as well as damaged telomeres to promote genomic stability^73^. In the developing brain, radiation leads to p53 binding to an alternative promoter of *Zfp365* that induces alternative splicing^70^. *Ano3* encodes Anoactamin3, a protein mainly expressed in neuronal tissues that functions as a K^+^ channel regulator^74^. Interestingly, mutations in human *ANO3* are associated with craniocervical dystonia, a neurological movement disorder^75^. *Ano3* is directly regulated by p53 and upregulated in response to radiation in the developing murine nervous system^70^. Although the exact contribution of these proteins to the testis phenotype is unknown, their dysregulation may suggest disrupted p53 signaling and cell cycle progression.

Several lncRNAs (*Gm614*, *Gm20904*, *Meg3* and *Malat1*) were found to be highly upregulated in *C* ^+/+^/*Tia1* ^−/−^ testis (Fig. 6D). *Meg3* is a tumor suppressor and *Malat1* regulates phosphorylation of a splicing factor^34^. Expression of both these lncRNAs was corrected in P60 testis upon early (P1 and P3) treatment with an antisense oligonucleotide targeted to *SMN2* intron 7^34^. Low SMN levels during early postnatal development might significantly affect RNA metabolism with implications for long-term testis health. The further dysregulation of lncRNA suggests that Tia1 may cooperate with SMN in RNA metabolism during testis development, and the loss of TIA1 further impairs this process. Taken together, the deletion of *Tia1* in the *C* ^+/+^ mouse appears to disrupt many physiological processes within the testis. The consequence of the complex interaction of these disturbed pathways is exacerbated degradation of the testis.

Disruption of ubiquitin homeostasis has been reported in the severe Taiwanese type I mice^76^. SMN interacts with UBA1, one of the enzymes required for the activation of ubiquitin for the downstream transfer to the target proteins by E3 ligases. Restoration of UBA1 ameliorated the SMA-like phenotype in mice and zebrafish^77^. As an E3 ligase, Herc6 is part of the ubiquitin pathway that promotes ubiquitination of target proteins for proteasome degradation. The finding that *Herc6* is downregulated in *Tia1* ^−/−^ brain and testis (Tables 1 and 2) suggests that Tia1 plays an important role in maintaining the ubiquitin homeostasis in these tissues. Our results are consistent with a recent report showing seminal vesicle hypertrophy in *Herc6* knockout mice^78^. Now that we have established a clear connection between SMN and TIA1 functions, future studies will determine the mechanism by which SMN and TIA1 collaborate to regulate the expression of various genes in different tissues.

The role of TIA1 has been implicated in several neurological diseases including ALS, Alzheimer’s disease and Frontotemporal Lobar Dementia^79^. Based on our findings that *Tia1* deletion differently affects gene expression in brain of males and females, we are tempted to speculate that TIA1 may serve as a gender-specific modifier of the above-mentioned neurological diseases. With significance to the heritable behavioral EEG-alpha trait, TIA1 has recently been implicated in epigenetic modification of the human genome^80^. The *C* ^+/+^/*Tia1* ^−/−^ model reported here provides an invaluable tool to address several fundamental questions including how the loss of *Tia1* combined with low SMN level impacts epigenetic modifications and temporally controls the transcriptional landscape in various tissues. The novel *C* ^+/+^/*Tia1* ^−/−^ model is also suited for testing the efficacy of therapeutic compounds aimed at the treatment of SMA and possibly other genetic diseases impacted by aberrant *SMN* and/or *TIA1* expression. To a broader significance, our findings set the stage for future studies aimed at uncovering the role of additional factors as gender-specific disease modifiers of SMA, one of the leading genetic diseases of children and infants.

## Materials and Methods

All methods were performed in accordance with approved biosafety and radiation safety guidelines for Iowa State University (ISU) and adhered to federal and state guidelines. All animal experiments were carried out following protocols approved by the Institutional Animal Care and Use Committee (IACUC) at ISU. All animal procedures adhered to the guidelines of the American Veterinary Medical Association (AVMA), United States Health and Human Services (US HHS), United States Department of Agriculture (USDA) and the State of Iowa.

### Generation of *C*^+/+^/*Tia1*^−/−^ mice

Mice hemizygous for the C allele (*Smn* ^+/−^/*C* ^+/−^)^32^ on the C57Bl/6 background was purchased from Jackson Laboratory (stock number 008714). Breeding pairs heterozygous for targeted *Tia1* knockout (*Tia1* ^+/−^)^29^, also on the C57Bl/6 background, were obtained from Paul Anderson’s laboratory at Harvard University. To generate first generation mice, a *Smn* ^+/−^/*C* ^+/−^/*Tia1* ^+/+^ mouse was mated with a *Smn* ^+/+^/*C* ^−/−^/*Tia1* ^+/−^ mouse. The desired mice from this cross were *Smn* ^+/−^/*C* ^+/−^/*Tia1* ^+/−^. Breeding pairs of these mice were used to generate mice of the specific genotype. However, because only one out of every sixteen offspring would be *C* ^+/+^/*Tia1* ^−/−^, we also established breeding colonies with both *Smn* ^+/−^/*C* ^+/−^/*Tia1* ^+/+^ parents or both *Smn* ^+/−^/*C* ^+/−^/*Tia1* ^−/−^ parents. Mice from all three breeding schemes were utilized for the subsequent studies. Records were kept on the number of pups born in each litter for each breeding pair.

### Measurement of weight and tail length during development

Pups from breeding cages with *Smn* ^+/−^/*C* ^+/−^/*Tia1* ^+/+^ parents or *Smn* ^+/−^/*C* ^+/−^/*Tia1* ^−/−^ parents were weighed and tails were measured every other day beginning on postnatal day 1 (P1) through P59. Each pup was marked with a color Sharpie™ on the belly and/or tail to differentiate pups in the same litter. After weaning, pups were separated by sex, identified with an ear tag and genotyped. Mice of the appropriate genotype were euthanized for subsequent studies at P60.

### Tissue collection

To assess histology, approximately six-week old mice of the appropriate genotype were weighed, deeply anesthetized with 100 mg/kg ketamine and 10 mg/kg xylazine and transcardially perfused with phosphate buffered saline (PBS) followed by 4% paraformaldehyde (pH 7.4). Following perfusion, the sex organs were removed, weighed with an analytical balance (Ohaus) and post-fixed overnight in modified Davidson’s fixative (Electron Microscopy Sciences) at 4 °C. The abdomen was opened and the entire mouse carcass was post-fixed and stored in neutral buffered formalin (Thermo Fisher Scientific) at room temperature. Subsequently, relevant tissues were dissected from the carcass, placed in cassettes, and submitted to the Histopathology Core in the Department of Veterinary Pathology at Iowa State University for processing, embedding in paraffin, and hematoxylin and eosin (H&E) staining. Tissues were processed with a modified, shorter ethanol-based run to better preserve tissue architecture. Each mouse was assigned a unique number to blind the experimenter from the genotype of each mouse.

To assess biochemical changes, a separate cohort of approximately six week old mice of the appropriate genotype were weighed and deeply anesthetized with 100 mg/kg ketamine and 10 mg/kg xylazine. The carotid artery was pierced to collect blood and the mouse was subsequently perfused with PBS to flush blood out of the tissues. Tissues were collected, frozen on dry ice and stored at −80 °C until further processing.

### Histological analysis of testis

H&E stained testis cross-sections were analyzed by a board-certified veterinary pathologist to determine any pathological changes. Photographs of sections were taken with a Nikon microscope with Spot Advanced software (Diagnostic Imaging, Inc.). Seminiferous tubules were analyzed with a previously published 10-point scoring system to assess the progression of spermatogenesis^36^. Twenty seminiferous tubules from all areas of each testis were scored. The average seminiferous tubule score was determined for each mouse and the distribution of scores for each genotype was also analyzed. The scoring was as follows:

1. No cells in tubular cross section
2. Sertoli cells only
3. Only spermatogonia present
4. No spermatozoa, no spermatids, but fewer than 5 spermatocytes present
5. No spermatozoa, no spermatids, but many spermatocytes present
6. No spermatozoa, but fewer than 5–10 spermatids present
7. 7No spermatozoa, but many spermatids present
8. All stages of spermatogenesis present, but fewer than 5–10 spermatozoa present
9. Many spermatozoa present, but germinal epithelium disorganized with marked sloughing or obliteration of lumen
10. Complete spermatogenesis

### Terminal deoxynucleotidyl transferase dUTP nick end labeling (TUNEL) staining

Three-μm formalin-fixed paraffin-embedded testis cross sections from P42 mice were subjected to TUNEL staining with the *In situ* Cell Death Detection Assay, Fluorescein (Roche) following the manufacturer’s protocol. For the negative control, two slides were incubated with only Labeling Solution (no enzyme). For the positive control, one slide was incubated with 2 U/μL DNase I, recombinant (Roche) for 10 minutes at room temperature to induce DNA fragmentation and then incubated as per the kit protocol. After labeling, sections were coverslipped with VectaShield Mounting Medium with DAPI (Vectastain) and imaged. Eight to ten micrographs from all regions of the testis were captured for analysis. TUNEL positive bodies were counted in at least 50 tubules from all regions of the testis for quantification.

### Epididymal sperm counting

P60 male mice of all genotypes were anesthetized with isoflurane and killed by cervical dislocation. The sex organs, including epididymides and testes, were dissected and removed. Epididymides were punctured with a fresh number 10 disposable scalpel and placed in 3 mL Dulbecco’s Modified Eagles’ Medium supplemented with 10% fetal bovine serum (Life Technologies) for 15 minutes at 37 °C. The cell suspension was triturated 15 times with a P1000 pipet. Two- and ten-fold dilutions of the cell suspension were prepared in water and incubated at room temperature for two minutes. The diluted cell suspension was loaded on a haemocytometer, allowed to settle, and sperm number counted. Only sperm with a head and tail were counted.

### Protein isolation, immunoblotting and densitometry

P42 testis was homogenized in ten volumes of radio immunoprecipitation assay (RIPA) buffer (Boston Biotechnologies) with 1X HALT protease inhibitor (Thermo Fisher Scientific) using a Microson Ultrasonic Cell Disrupter set at speed 2 (Misonix). The homogenate was incubated on ice for 30 minutes and then centrifuged at 12,000 × g for 15 minutes at 4 °C and the supernatant transferred to a new tube. Protein concentration was determined with the Protein Dye Assay Concentrate (BioRad).

Protein lysates were diluted with 2X Laemlli sample buffer (BioRad) with 10% β-mercaptoethanol (Sigma-Aldrich). Samples were boiled for five minutes and then centrifuged at maximum speed for five minutes at room temperature. Fifteen µg protein was separated on 10% SDS-PAGE gels. The separated protein was transferred to Immobilon polyvinylidene fluoride (PVDF) membrane (Millipore) using the Trans-Blot Turbo system (BioRad). Membranes were incubated in 5% nonfat milk (Walmart) prepared in 1X Tris-buffered saline with Tween 20 (TBST; 50 mM Tris; 150 mM NaCl, 0.05% Tween-20) and subsequently incubated in primary antibody. Primary antibodies were: mouse anti-SMN (BD Biosciences; 610646) diluted 1:2000; donkey anti-TIA1 (Santa Cruz Biotechnologies; sc-1751) diluted 1:250; donkey anti-TIAR (Santa Cruz Biotechnolgies; sc-1749) diluted 1:250; mouse anti-Gemin2 clone 2E17 (Sigma; G6669) diluted 1:750; rabbit anti-β-actin (Sigma; A2103) diluted 1:2000. Primary incubations were performed either overnight at 4 °C with shaking (anti-SMN, TIA1, TIAR and Gemin2) or for 1 hour at room temperature (β-actin). The blots were then washed with TBST and incubated in secondary antibody diluted 1:4000 in 5% milk for 1 hour at room temperature. Secondary antibodies were: goat anti-mouse IgG (Jackson Immunoresearch; 115-035-003); donkey anti-goat IgG (Santa Cruz Biotechnologies; sc-2020) or donkey anti-rabbit IgG (GE Healhcare; NA934V) all conjugated to horseradish peroxidase (HRP). Protein was detected using WestFemto substrate (Thermo Fisher Scientific) and visualized with the BioSpectrum AC Imaging System (UVP). For each blot, the mean intensity of each band was determined using ImageJ software. The SMN, Tia1, Tiar or Gemin2 mean intensities were then divided by the β-actin mean intensity for the same lane to normalize the data and expressed as a percent of WT.

### Testosterone enzyme linked immunosorbent assay (ELISA)

Total serum testosterone was measured using the Mouse/Rat Testosterone ELISA kit (CalBiotech) using the manufacturer’s instructions. A standard curve was constructed using GraphPad Prism v6.0 using a sigmoidal 4-parameter logistics fit and testosterone concentration (ng/ml) were determined based on this curve.

### RNA-seq analysis of mouse transcriptome

RNA was isolated from P42 testis, liver, and brain (n = 4 for all genotypes and tissues except for C/C testis, n = 2 for C/C testis; liver and brain consisted of 2 males and 2 females per genotype) using TRIzol reagent according to the manufacturer’s instructions. Library preparation, sequencing, and read quality control were performed as previously described^33^. Briefly, RNA was treated with RQ1 RNase-free DNase (Promega), repurified, and quantified/characterized using an Agilent Bioanalyzer (Agilent Technologies). RNA was poly(A) enriched and libraries prepared using the NEBNext Ultra Directional Library Prep Kit (New England Biolabs). Libraries were sequenced on an Illumina HiSeq. 2500. Reads were trimmed and low-quality reads removed using cutadapt and mapped to the GRm38/mm10 genome using Tophat. Read counts were generated on a per-gene basis using HTSeq-count. To identify differentially expressed genes, read counts for all samples were imported into DESeq. 2 simultaneously for each tissue and pairwise comparisons were made by specifying the “contrast” parameter within the “results” command. KEGG pathway analysis was performed using theWebGESTALT web server (http://bioinfo.vanderbilt.edu/webgestalt/).

### Quantitative real-time PCR (QPCR) and multi-exon skipping detection assay (MESDA)

Total RNA was isolated from a hemibrain or whole testis using TRIzol reagent (Thermo Fisher) following the manufacturer’s protocol. A portion of the isolated RNA was subjected to DNase treatment with RQ1 RNase-free DNase (Promega) following the manufacturer’s protocol. RNA was cleaned up using the RNeasy Mini kit (Qiagen) and RNA concentration was determined. One µg (testis) or 1.25 µg (brain) of RNA was reverse transcribed in a 10 µL reaction using the SuperScript III reverse transcription kit (Thermo Fisher Scientific) with Random Primers (Promega; 0.25 µg per reaction). The cDNA was further diluted 20-fold (testis) or 25-fold (brain) with RNase-free water. QPCR was performed using a Stratagene Mx3000P or Mx3005 QPCR system (Agilent Technologies) and the FastStart Universal SYBR Green Master Mix with Rox (Roche). Primer sequences are listed in Supplementary Table 10. Each reaction was performed with ~10 ng cDNA. *Gapdh* was used as the housekeeping gene for normalization. Data was analyzed using the ΔΔCt method with WT set as the reference condition.

MESDA was performed as described previously^33, 35^. Briefly, DNase-treated RNA was reverse transcribed in a 5 µL reaction using 3′E8-DdeI (CTACAACACCCTTCTCACAGCTC) primer (0.5 pmol per reaction). PCR was performed using 5′E1 (CGCGGGTTTGCTATGGCGAT) and 3′E8-25 (TTAGTGCTGCTCTATGCCAGCATTT) primers to amplify in the presence of 3′E8-25 primer that was labeled at the 5′ end with [γ-^32^P] ATP (6000 Ci/mmol) (Perkin Elmer). PCR products were run on 4% native polyacrylamide gels, which were then dried and exposed to a phosphorimager screen. Gel images were scanned using a Fujifilm FLA-5100 and densitometric quantification was carried out using Multi Gauge software (Fujifilm).

### Statistical analysis

All statistical analysis was performed with GraphPad Prism v6.0. All data are presented as mean ± standard error of the mean (S.E.M.). For all analysis, a *p* value less than 0.05 was considered significant. Weight and tail measurements over time were analyzed with repeated measures two-way analysis of variance (ANOVA) with Tukey’s multiple comparison test to determine differences between genotypes. Data on percent weight gain, maximum tail length, male and female reproductive measures, densitometry and gene expression changes were compared between genotypes using a one-way ANOVA with Tukey’s multiple comparison test. The results of Tukey’s multiple comparison test are denoted in the graphs and tables by lowercase letters. For graphs, bars that have different letters represent a statistically significant difference between the means, whereas bars that have the same letters are not significantly different. For the tables, mean ± SEM values for the same gene that have different superscript letters are significantly different, whereas values with same letters are not different. Data on age of visible tail or ear necrosis onset was compared between genotypes using an unpaired two-tailed Student’s *t* test.

### Data Availability

The RNA-seq data generated during the current study are available in the Sequence Read Archive under accession number SRP106484 (https://www.ncbi.nlm.nih.gov/sra/?term=SRP106484).

## Electronic supplementary material

Supplementary Material
