# Supplementary material for: TIA1 is a gender-specific disease modifier of a mild mouse model of spinal muscular atrophy
Source: Sci Rep. 2017 Aug 3;7:7183. doi: 10.1038/s41598-017-07468-2 (PMC5543135; doi:10.1038/s41598-017-07468-2)
Supplement: Supplementary file 1 — Supplementary Material [file 41598_2017_7468_MOESM1_ESM.pdf]

TIA1 is a gender-specific disease modifier of a mild mouse model of spinal muscular atrophy

Matthew D. Howell, Eric W. Ottesen, Natalia N. Singh, Rachel L. Anderson, Joonbae Seo, Senthilkumar Sivanesan, Elizabeth M. Whitley, Ravindra N. Singh

Supplementary Figures, Tables and Methods

Contents:

Supplementary Figures (8)

Supplementary Tables (10)

Supplementary Methods

Supplementary Figure Legends

Supplementary Fig. 1: Full-length western blots for testis protein (from Fig. 3K). The antibody used to probe each blot is listed in the upper left corner. Molecular weights are noted to the left of each blot. Genotypes and lane numbers are listed above and below each blot, respectively.

Supplementary Fig. 2: P42 $C^{+/+}$ and $C^{+/+}$/*Tia1*$^{-/-}$ female reproductive organs do not display abnormalities. (A) Photograph of representative uterus and ovaries from P42 females of each genotype. Scale bar is 10 mm. (B) Gross uterus and ovaries mass for P42 mice. (C) Relative uterus and ovaries mass for P42 mice was determined by dividing the total uterus and ovaries mass by the total body weight. For (B) and (C), n=10, 10, 7 and 9 WT, *Tia1*$^{-/-}$, $C^{+/+}$ and $C^{+/+}$/*Tia1*$^{-/-}$ mice, respectively. (D) Representative micrographs of H&E cross sections of the uterine endometrium (top) and ovary (bottom). Genotypes are indicated at the top of each micrograph. Scale bar is 100 μm. (E) Representative western blots for SMN, Gemin2, Tia1, Tiar and loading control β-actin proteins in P42 uterus and ovaries. Full-length blots are presented in Supplementary Fig. 3. Genotypes and lane numbers are indicated above and below the blots, respectively. The graphs to the right indicate the results of densitometry (n=4 mice per genotype). Error bars on all graphs indicate S.E.M. For (B), (C) and (E), statistical comparison between genotypes was performed using a one-way ANOVA followed by Tukey's multiple comparison test and the results are denoted by lowercase letters. Bars with different letters indicate a statistically significant different between the means, whereas bars with the same letters

are not significantly different (p<0.05). For (B) and (C), data from WT and $C^{+/+}$ mice was previously published[33].

Supplementary Fig. 3: Full-length western blots for uterus/ovaries protein (from Supplementary Fig. 2E). The antibody used to probe each blot is listed in the upper left corner. Molecular weights are noted to the left of each blot. Genotypes and lane numbers are listed above and below each, respectively.

Supplementary Fig. 4: Protein expression in P42 brain, spinal cord or heart is not different between $C^{+/+}$ and $C^{+/+}$/*Tia1*$^{-/-}$ males or females. (A) Representative western blots for SMN, Gemin2, Tia1, Tiar and loading control β-actin proteins in P42 male and female brain. Full-length blots are presented in Supplementary Fig. 5. Genotypes and lane numbers are indicated above and below the blots, respectively. The graphs to the right indicate the results of densitometry (n=4 mice per genotype and sex). (B) Representative western blots for SMN, Gemin2, Tia1, Tiar and loading control β-actin proteins in P42 male and female spinal cord. Full-length blots are presented in Supplementary Fig. 6. Genotypes and lane numbers are indicated above and below the blots, respectively. The graphs to the right indicate the results of densitometry (n=4 mice per genotype and sex). (C) Representative western blots for SMN, Gemin2, Tia1, Tiar and loading control β-actin proteins in P42 male and female heart. Full-length blots are presented in Supplementary Fig. 7. Genotypes and lanes numbers are indicated above and below the blots, respectively. The graphs to the right indicate the results of densitometry (n=4 mice per genotype and sex). Error bars on all graphs indicate S.E.M. For each tissue and sex, statistical comparison between the genotypes was performed using a one-way

ANOVA followed by Tukey's multiple comparison test and the results are denoted by lowercase letters. Bars with different letters indicate a statistically significant different between the means, whereas bars with the same letters are not significantly different (p<0.05).

Supplementary Fig. 5: Full-length western blots for male and female brain protein (from Supplementary Fig. 4A). The antibody used to probe each blot and the sex are listed in the upper left corner. Molecular weights are noted to the left of each blot. Genotypes and lane numbers are listed above and below each blot, respectively.

Supplementary Fig. 6: Full-length western blots for male and female spinal cord protein (from Supplementary Fig. 4B). The antibody used to probe each blot and the sex are listed in the upper left corner. Molecular weights are noted to the left of each blot. Genotypes and lane numbers are listed above and below each blot, respectively.

Supplementary Fig. 7: Full-length western blots for male and female heart protein (from Supplementary Fig. 4C). The antibody used to probe each blot the sex are listed in the upper left corner. Molecular weights are noted to the left of each blot. Genotypes and lane numbers are listed above and below the blots, respectively.

Supplementary Fig. 8: Reduction of *Smn* and *Tia1* knockout alone or in combination only mildly affects the brain and liver transcriptome. (A) MA plots of estimated gene expression in brain for each mutant genotype compared to WT (top row) and $C^{+/+}$/$Tia1^{-/-}$ compared to each single mutant (bottom row). The y-axis depicts $\log_2$ fold change (L2FC) in gene expression in

each comparison, and the x-axis depicts the mean read count for each gene between all samples. Each dot represents one gene, with red dots representing genes with significantly altered expression values (Benjamini and Hochberg (B+H) adjusted p value < 0.05). (B) MA plots of estimated gene expression in liver for each mutant genotype compared to WT (top row) and $C^{+/+}/Tia1^{-/-}$ compared to each single mutant (bottom row) in. Details about the graphs are the same as in (A).

Supplementary Fig. 1

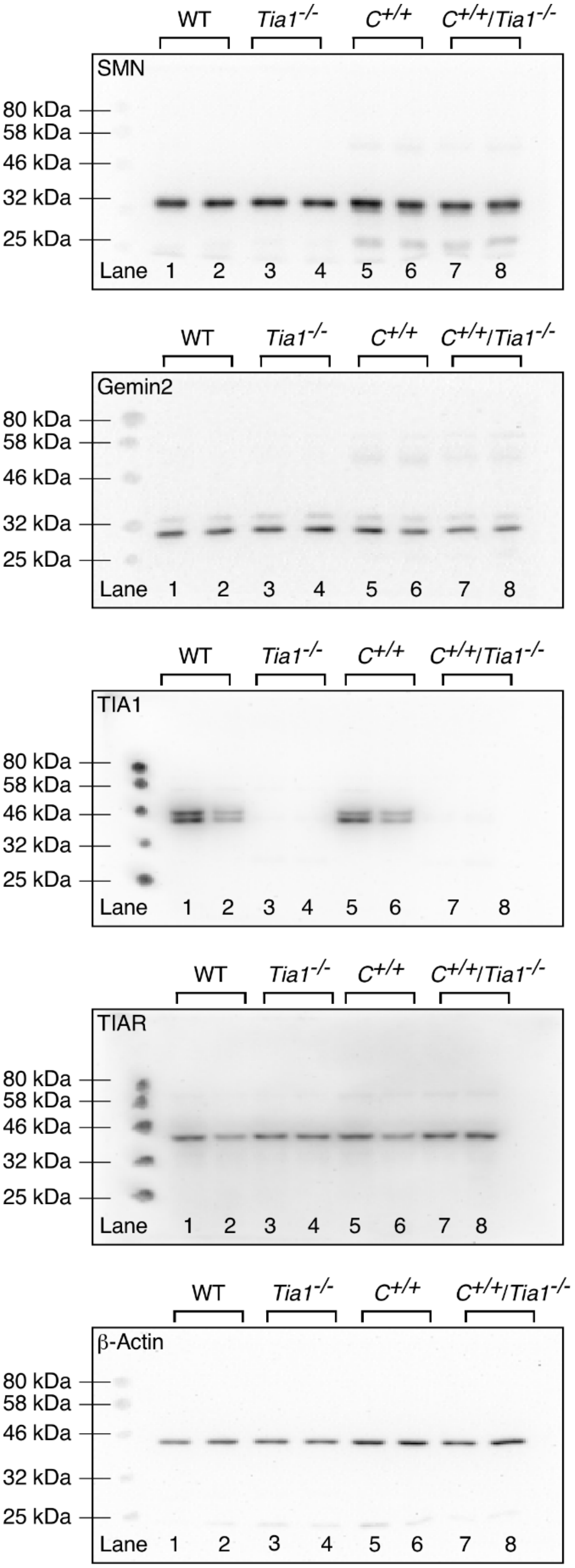

Supplementary Fig. 2

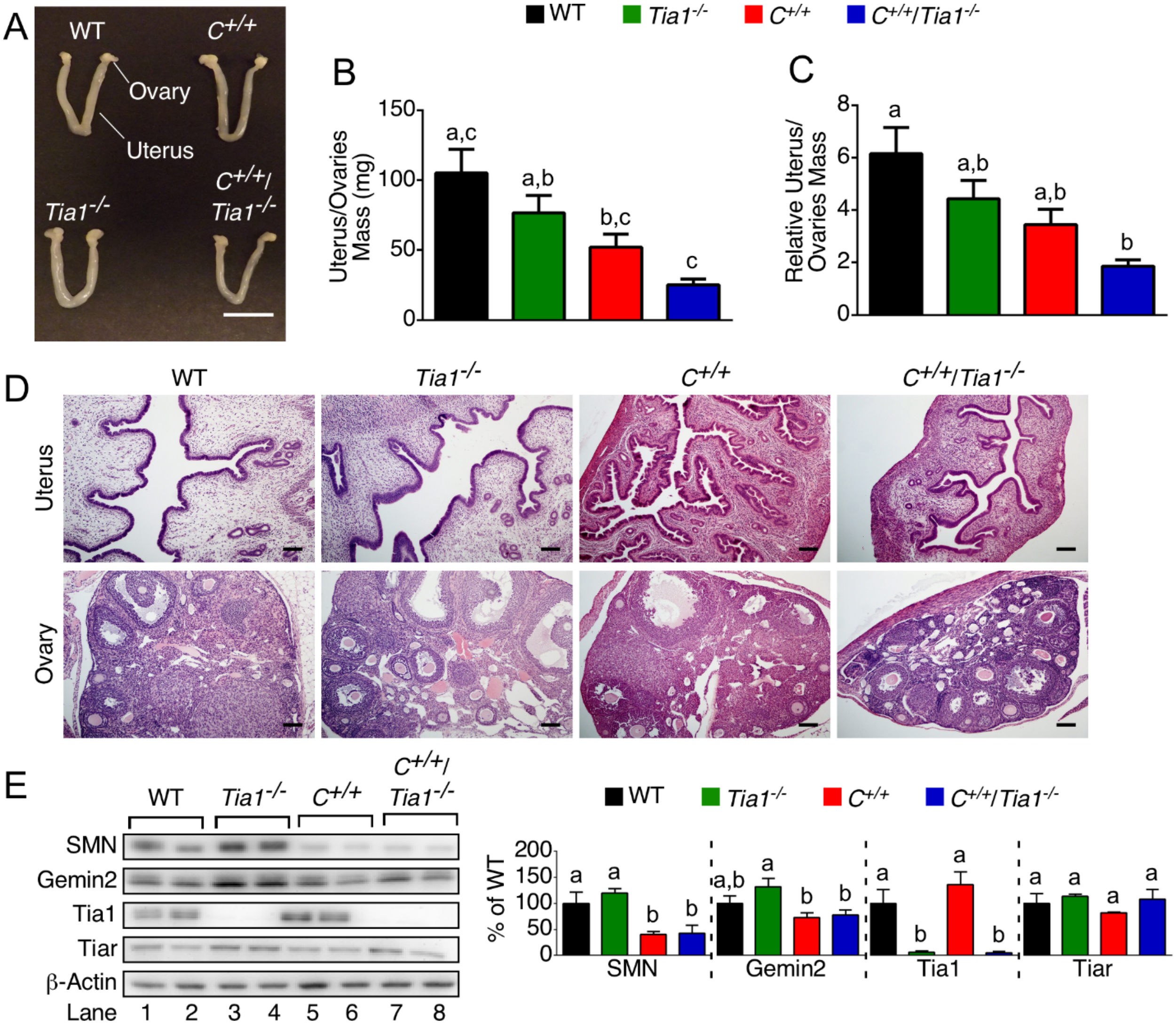

Supplementary Fig. 3

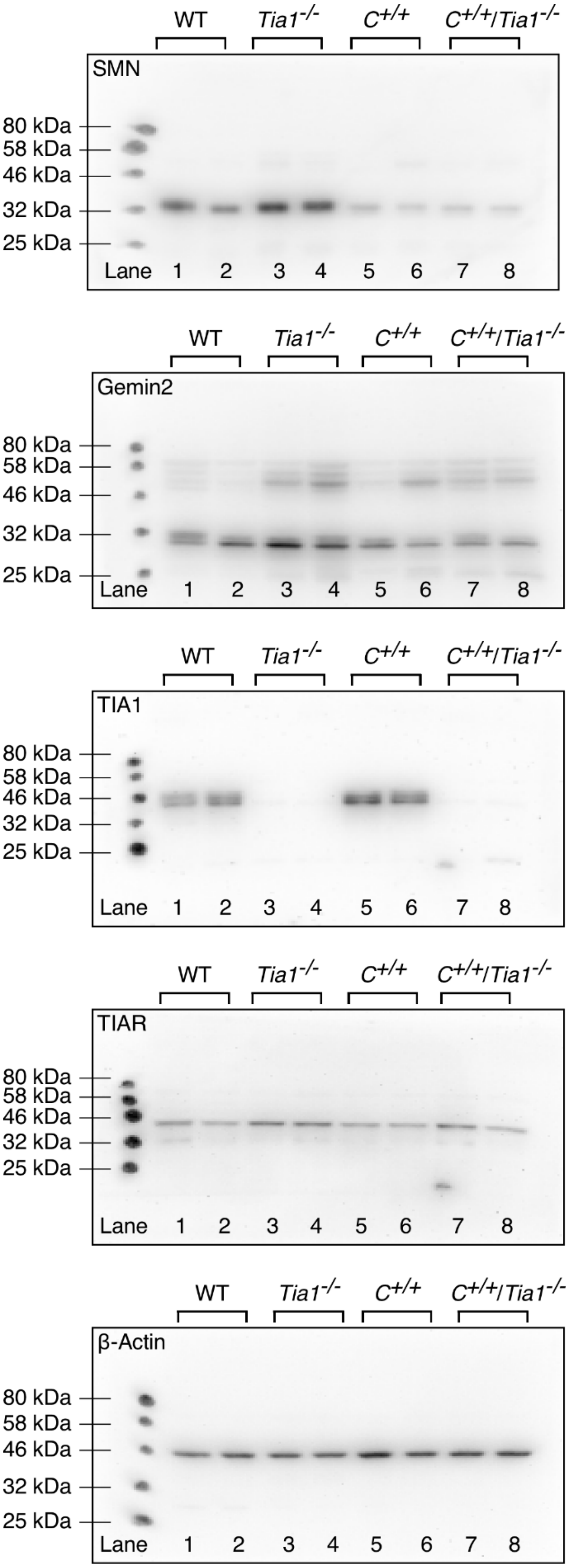

Supplementary Fig. 4

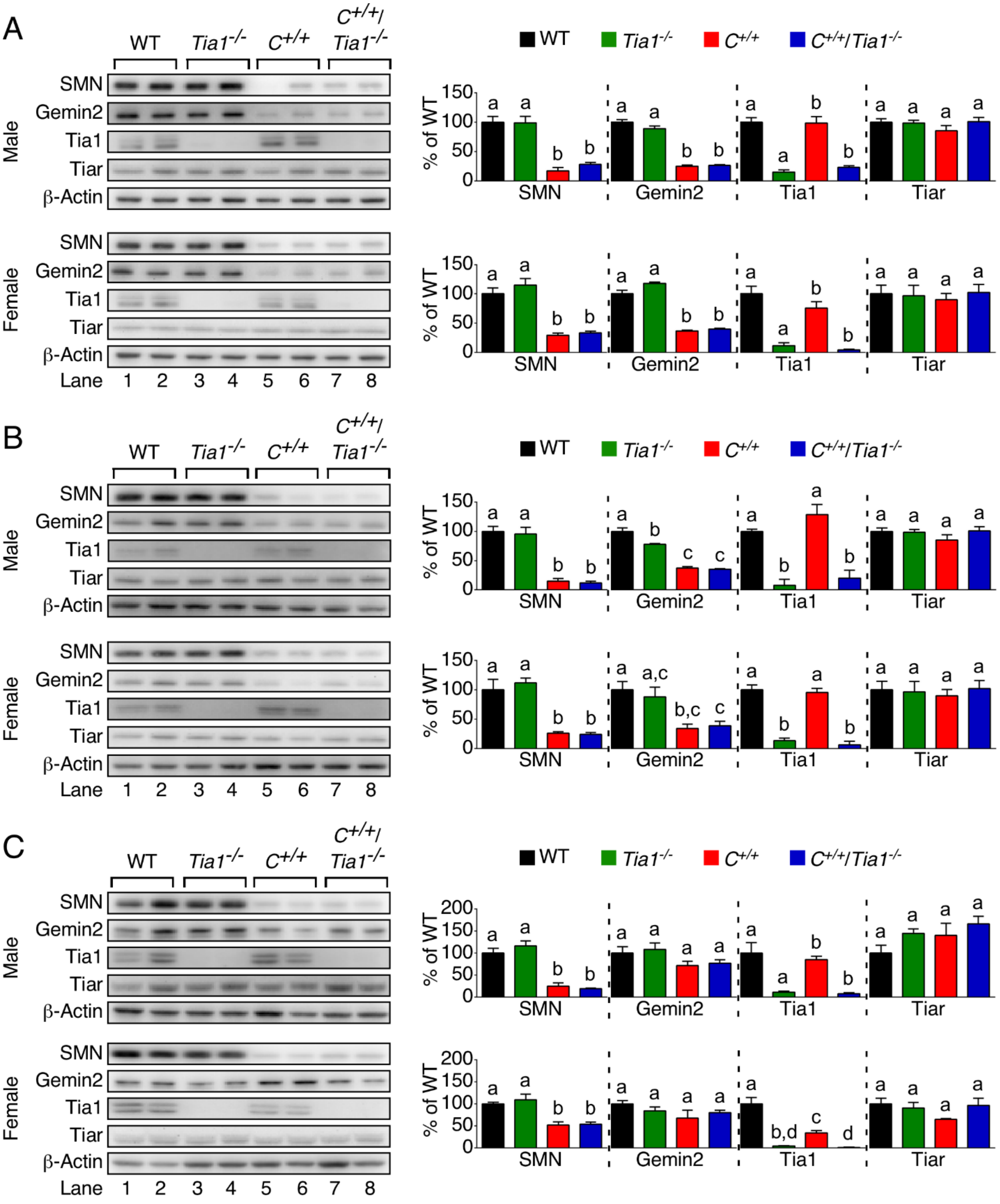

Supplementary Fig. 5

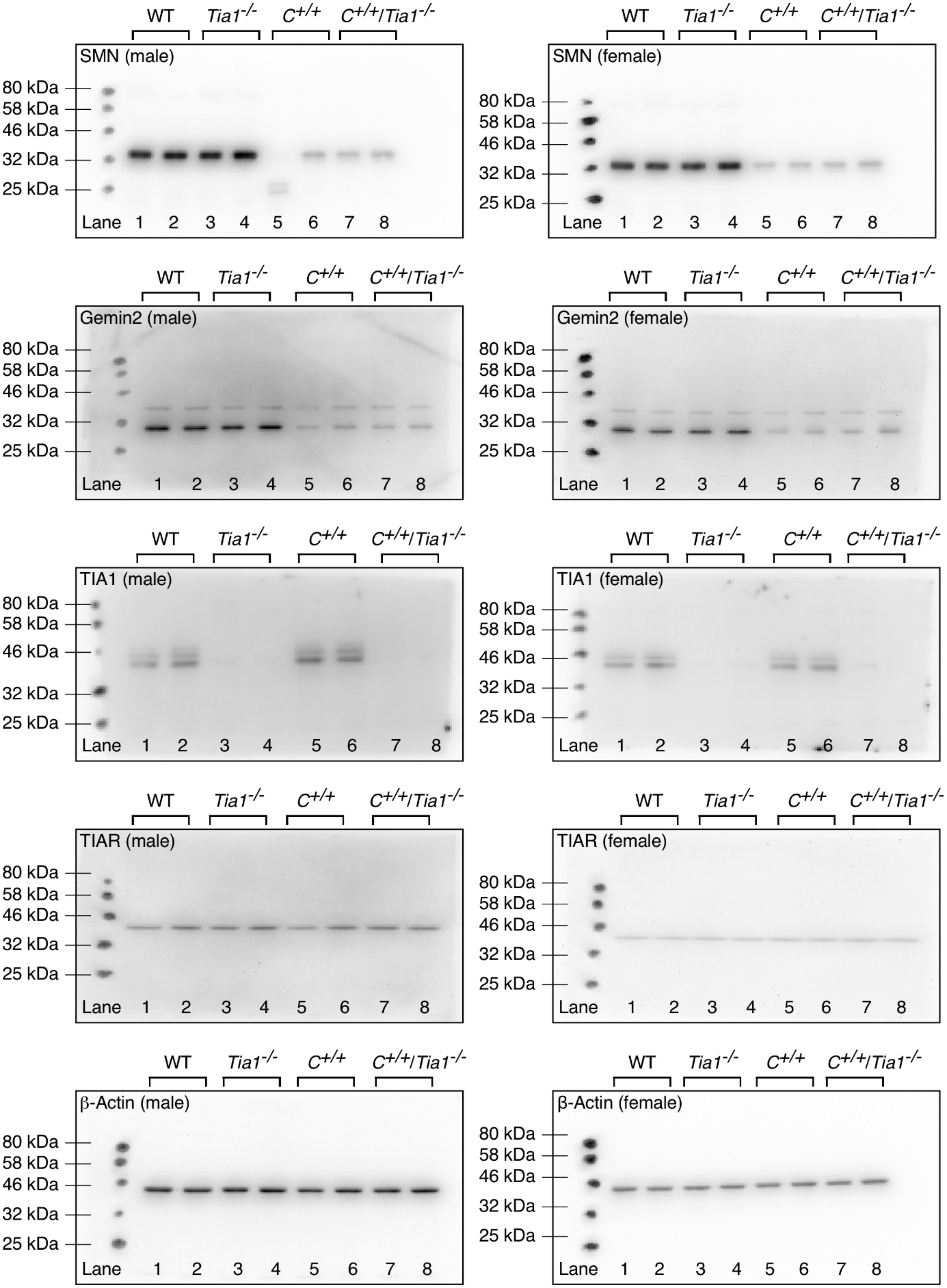

Supplementary Fig. 6

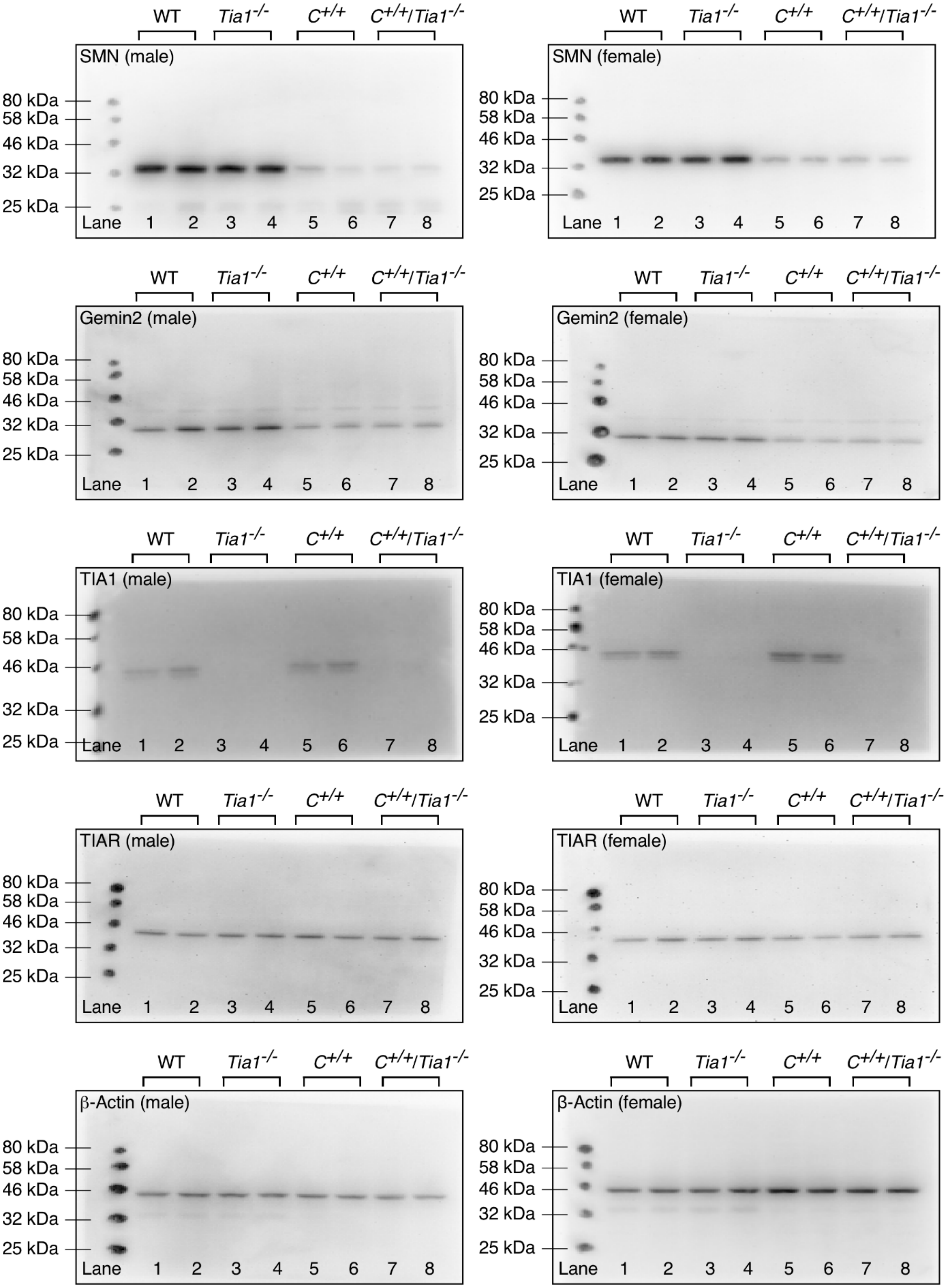

Supplementary Fig. 7

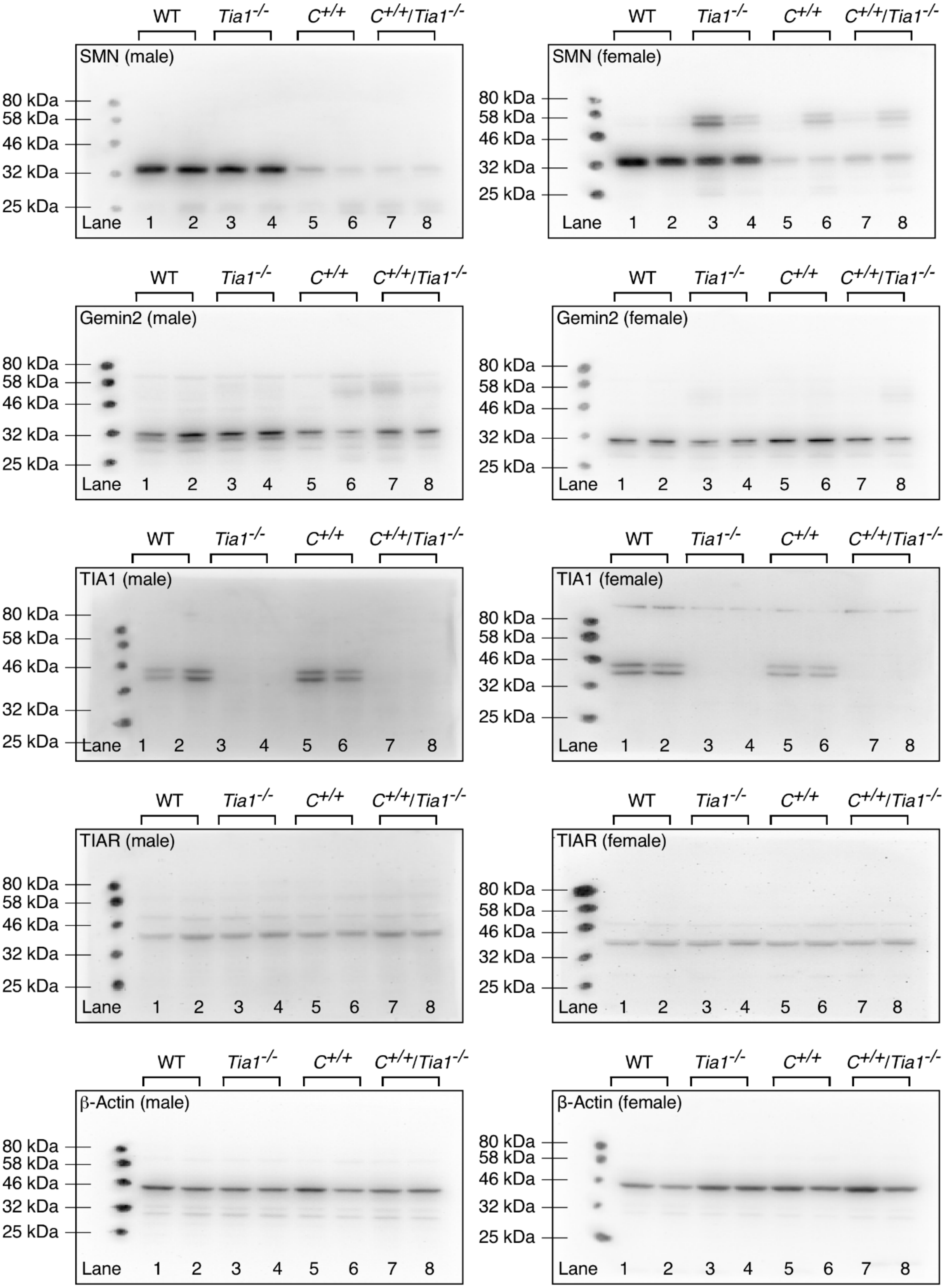

Supplementary Fig. 8

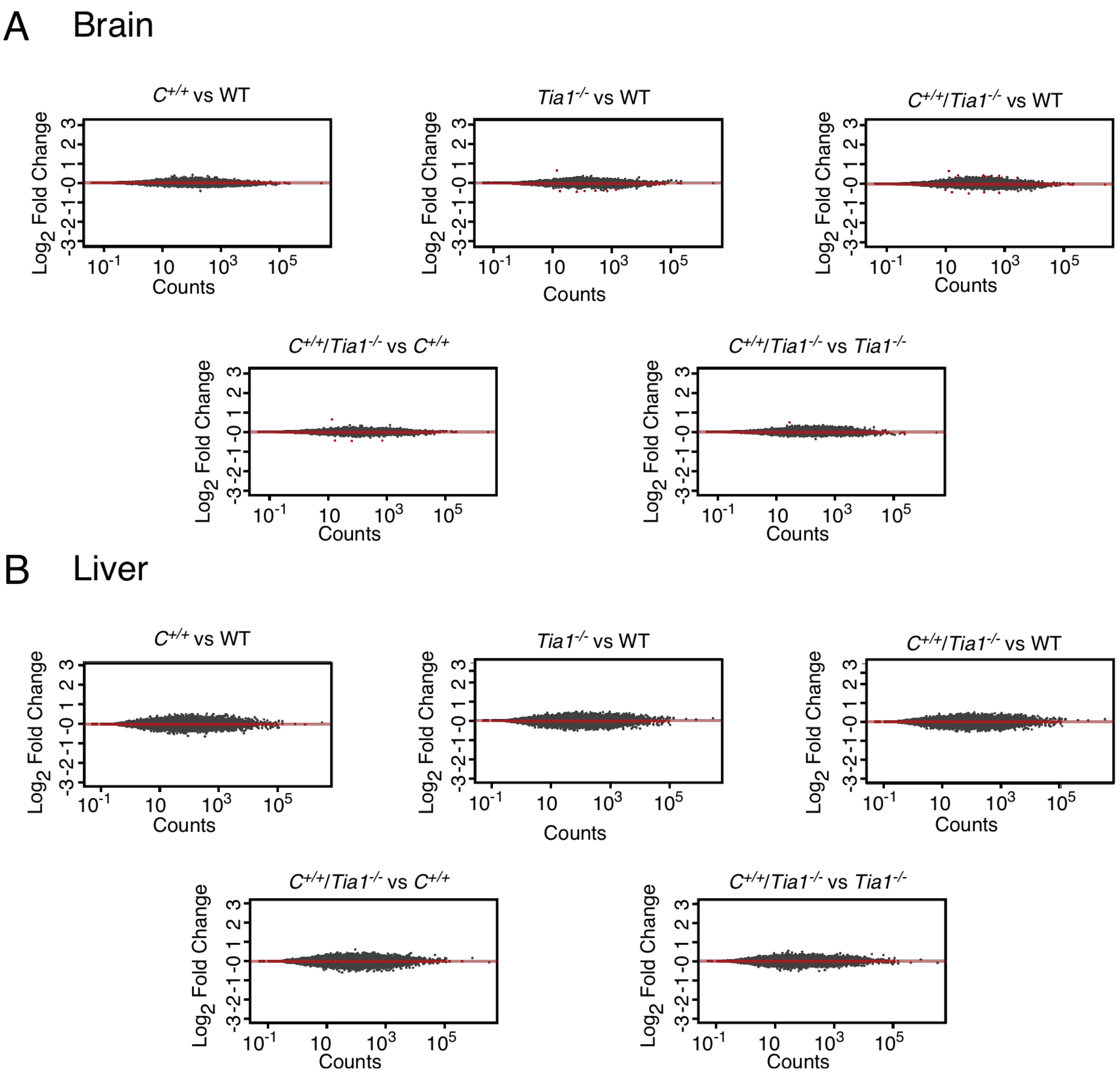

Supplementary Tables

Supplementary Table 1: Observed and expected offspring *Tia1* genotypes

**Offspring *Tia1* Genotypes**	**Breeding Scheme**	
	***Smn*$^{+/-}$/*C*$^{+/-}$/*Tia1*$^{+/-}$ (Male); *Smn*$^{+/-}$/*C*$^{+/-}$/*Tia1*$^{+/-}$ (Female)**	
	**Observed**	**Expected**
Tia1$^{+/+}$	45	44
Tia1$^{+/-}$	81	88
Tia1$^{-/-}$	50	44
χ^2 value	0.6842	
p value	0.7103	

Expected number of pups from matings based on total number of pups born (25% *Tia1*$^{+/+}$, 50% *Tia1*$^{+/-}$ and 25% *Tia1*$^{-/-}$)

Supplementary Table 2: Observed and expected offspring genotypes from breeding schemes

Offspring Genotypes	Breeding Scheme					
	***Smn$^{+/-}$*/C$^{+/-}$/*Tia1$^{+/+}$* (Male); *Smn$^{+/-}$/C$^{+/-}$/ Tia1$^{+/+}$* (Female)**		***Smn$^{+/-}$/C$^{+/-}$/Tia1$^{+/-}$* (Male); *Smn$^{+/-}$/C$^{+/-}$/ Tia1$^{+/-}$* (Female)**		***Smn$^{+/-}$/C$^{+/-}$/Tia1$^{-/-}$* (Male); *Smn$^{+/-}$/C$^{+/-}$/ Tia1$^{-/-}$* (Female)**	
	**Observed**	**Expected**	**Observed**	**Expected**	**Observed**	**Expected**
Smn$^{+/+}$/C$^{-/-}$/ Tia1$^{+/+}$[a]	49	46	14	11	-	-
Smn$^{+/+}$/C$^{-/-}$/ Tia1$^{+/-}$	-	-	26	22	-	-
Smn$^{+/+}$/C$^{-/-}$/ Tia1$^{-/-}$ [b]	-	-	12	11	49	45
Smn$^{+/-}$/C$^{+/-}$/ Tia1$^{+/+}$	95	91	22	22	-	-
Smn$^{+/-}$/C$^{+/-}$/ Tia1$^{+/-}$	-	-	37	44	-	-
Smn$^{+/-}$/C$^{+/-}$/ Tia1$^{-/-}$	-	-	25	22	91	90
Smn$^{-/-}$/C$^{+/+}$/ Tia1$^{+/+}$ [c]	39	46	9	11	-	-
Smn$^{-/-}$/C$^{+/+}$/ Tia1$^{+/-}$	-	-	18	22	-	-
Smn$^{-/-}$/C$^{+/+}$/ Tia1$^{-/-}$ [d]	-	-	13	11	41	45
χ^2 value	0.7572		2.3		0.359	
p value	0.6848		0.9704		0.8357	

[a] WT mice; [b] *Tia1$^{-/-}$* mice; [c] *C$^{+/+}$* mice; [d] *C$^{+/+}$/Tia1$^{-/-}$* mice

Expected numbers of pups based on total number of pups born. For breeding schemes where breeders are *Smn$^{+/-}$*/C$^{+/-}$/*Tia1$^{+/+}$* or *Smn$^{+/-}$*/C$^{+/-}$/*Tia1$^{-/-}$*, offspring *Smn* genotype is expected to be 25% *Smn$^{+/+}$/C$^{-/-}$*, 50% *Smn$^{+/-}$/C$^{+/-}$* and 25% *Smn$^{-/-}$/C$^{+/+}$*.

For breeding scheme where breeders are *Smn$^{+/-}$*/C$^{+/-}$/*Tia1$^{+/-}$*, offspring genotype is expected to be 6.25% *Smn$^{+/+}$/C$^{-/-}$/ Tia1$^{+/+}$*, 12.5% *Smn$^{+/+}$/C$^{-/-}$/ Tia1$^{+/-}$*, 6.25% *Smn$^{+/+}$/C$^{-/-}$/ Tia1$^{-/-}$*, 12.5% *Smn$^{+/-}$/C$^{+/-}$/ Tia1$^{+/+}$*, 25% *Smn$^{+/-}$/C$^{+/-}$/ Tia1$^{+/-}$*, 12.5% *Smn$^{+/-}$/C$^{+/-}$/ Tia1$^{-/-}$*, 6.25% *Smn$^{-/-}$/C$^{+/+}$/ Tia1$^{+/+}$*, 12.5% *Smn$^{-/-}$/C$^{+/+}$/Tia1$^{+/-}$* and 6.25% *Smn$^{-/-}$/C$^{+/+}$/Tia1$^{-/-}$*.

Supplementary Table 3: RNA-seq libraries and mapping statistics

Tissue	Mouse Identifier	Sex	Genotype	Index Sequence	Valid Reads	Mapped Reads	Mapping %
Brain	817	Male	WT	ATCACG	37433512	32458389	86.7%
	841	Male	WT	GATCAG	32324511	28435032	88.0%
	1241	Female	WT	CGATGT	31571131	27405557	86.8%
	1244	Female	WT	TAGCTT	36174505	33440900	92.4%
	1387	Male	$Tia1^{-/-}$	TTAGGC	34948333	29838886	85.4%
	1386	Male	$Tia1^{-/-}$	GGCTAC	25428906	24037618	94.5%
	1215	Female	$Tia1^{-/-}$	TGACCA	39102155	32930186	84.2%
	1298	Female	$Tia1^{-/-}$	CTTGTA	29948581	28367558	94.7%
	1395	Male	$C^{+/+}$	ACAGTG	19123835	16943311	88.6%
	818	Male	$C^{+/+}$	ATCACG	31980917	30267623	94.6%
	827	Female	$C^{+/+}$	GCCAAT	27106907	23679935	87.4%
	833	Female	$C^{+/+}$	CGATGT	29094629	27692886	95.2%
	1292	Male	$C^{+/+}/Tia1^{-/-}$	CAGATC	33589165	29630030	88.2%
	1213	Male	$C^{+/+}/Tia1^{-/}$	TTAGGC	31085675	29529727	95.0%
	1295	Female	$C^{+/+}/Tia1^{-/}$	ACTTGA	32494498	28685271	88.3%
	1210	Female	$C^{+/+}/Tia1^{-/}$	TGACCA	31239337	29599137	94.7%
Liver	841	Male	WT	ATCACG	25287387	20198643	79.9%
	1218	Male	WT	GATCAG	28848358	23031795	79.8%
	826	Female	WT	CGATGT	25290844	20348798	80.5%
	1241	Female	WT	GGCTAC	20849401	16929086	81.2%
	1030	Male	$Tia1^{-/-}$	TTAGGC	25386232	20323799	80.1%
	1388	Male	$Tia1^{-/-}$	TAGCTT	26637794	20704116	77.7%
	1079	Female	$Tia1^{-/-}$	TGACCA	27128959	21956200	80.9%
	1298	Female	$Tia1^{-/-}$	CTTGTA	27341080	21622431	79.1%
	818	Male	$C^{+/+}$	ACAGTG	26696226	21229870	79.5%
	839	Male	$C^{+/+}$	ATCACG	27076573	21975056	81.2%
	827	Female	$C^{+/+}$	GCCAAT	28782505	23028425	80.0%
	1097	Female	$C^{+/+}$	CGATGT	25268550	20401255	80.7%
	1213	Male	$C^{+/+}/Tia1^{-/}$	CAGATC	28471090	22531348	79.1%
	1292	Male	$C^{+/+}/Tia1^{-/}$	TTAGGC	28306284	22950010	81.1%
	1205	Female	$C^{+/+}/Tia1^{-/}$	ACTTGA	29688454	24241744	81.7%
	1296	Female	$C^{+/+}/Tia1^{-/}$	TGACCA	27093989	21649675	79.9%
Testis	817	Male	WT	ACAGTG	30537363	27333103	89.5%
	841	Male	WT	ATCACG	26659140	23366701	87.6%
	1218	Male	WT	GCCAAT	32045460	27572302	86.0%
	1222	Male	WT	CGATGT	23746444	20461630	86.2%
	1030	Male	$Tia1^{-/-}$	CAGATC	29299261	25593910	87.4%
	1033	Male	$Tia1^{-/-}$	TTAGGC	23535766	20877689	88.7%
	1387	Male	$Tia1^{-/-}$	ACTTGA	32051013	29014410	90.5%
	1392	Male	$Tia1^{-/-}$	TGACCA	24247222	22068514	91.0%
	839	Male	$C^{+/+}$	ACAGTG	23487896	21499042	91.5%
	1395	Male	$C^{+/+}$	TAGCTT	30248870	26555454	87.8%
	1075	Male	$C^{+/+}/Tia1^{-/}$	GCCAAT	25127307	23353423	92.9%
	1212	Male	$C^{+/+}/Tia1^{-/}$	GGCTAC	27844938	24617115	88.4%
	1213	Male	$C^{+/+}/Tia1^{-/}$	CAGATC	24528628	22150662	90.3%
	1292	Male	$C^{+/+}/Tia1^{-/}$	CTTGTA	31085633	28524790	91.8%

Supplementary Table 4: Significantly altered genes in mutant brain

Genotype Comparison	Accession	Gene Symbol	L2FC	Adjusted *p* value
Tia1$^{-/-}$ vs. WT	ENSMUSG00000022185	*Acin1*	-0.262	2.84E-02
	ENSMUSG00000029798	*Herc6*	-0.407	2.22E-02
	ENSMUSG00000043162	*Pyurf*	-0.395	9.29E-03
	ENSMUSG00000062190	*Lancl2*	-0.340	2.05E-02
	ENSMUSG00000064293	*Cntn4*	-0.418	2.86E-02
	ENSMUSG00000068303	*Spr-ps1*	0.660	3.38E-12
	ENSMUSG00000079494	*Nat8f5*	-0.421	5.34E-03
	ENSMUSG00000085067	*Gm15631*	-0.453	6.43E-03
C$^{+/+}$/*Tia1*$^{-/-}$ vs. WT	ENSMUSG00000015843	*Rxrg*	0.368	1.84E-02
	ENSMUSG00000018102	*Hist1h2bc*	0.410	2.80E-02
	ENSMUSG00000021645	*Smn1*	-0.439	3.11E-03
	ENSMUSG00000029608	*Rph3a*	-0.245	2.21E-02
	ENSMUSG00000029998	*Pcyox1*	-0.289	3.09E-03
	ENSMUSG00000030770	*Parva*	-0.284	2.21E-02
	ENSMUSG00000035226	*Rims4*	-0.269	1.70E-02
	ENSMUSG00000038805	*Six3*	0.381	1.08E-02
	ENSMUSG00000041730	*Prrxl1*	-0.322	6.18E-03
	ENSMUSG00000060981	*Hist1h4h*	0.428	3.61E-03
	ENSMUSG00000064293	*Cntn4*	-0.463	3.11E-03
	ENSMUSG00000068303	*Spr-ps1*	0.663	2.61E-12
	ENSMUSG00000068696	*Gpr88*	0.319	6.18E-03
	ENSMUSG00000079494	*Nat8f5*	-0.433	1.40E-03
	ENSMUSG00000079662	*Ntn3*	0.425	1.87E-02
	ENSMUSG00000082286	*Pisd-ps1*	0.416	2.21E-02
	ENSMUSG00000085067	*Gm15631*	-0.502	4.16E-04
C$^{+/+}$/*Tia1*$^{-/-}$ vs. *C*$^{+/+}$	ENSMUSG00000064293	*Cntn4*	-0.447	1.28E-02
	ENSMUSG00000068303	*Spr-ps1*	0.648	1.03E-11
	ENSMUSG00000079494	*Nat8f5*	-0.445	1.07E-03
	ENSMUSG00000085067	*Gm15631*	-0.467	2.64E-03
C$^{+/+}$/*Tia1*$^{-/-}$ vs. *Tia1*$^{-/-}$	ENSMUSG00000060981	*Hist1h4h*	0.494	5.99E-04

Supplementary Table 5: Significantly altered genes in mutant testis

Genotype Comparison	Accession	Gene Symbol	L2FC	Adjusted *p* value
Tia1$^{-/-}$ vs. WT	ENSMUSG00000034744	*Nagk*	0.659	4.76E-06
	ENSMUSG00000029798	*Herc6*	-1.161	6.47E-06
	ENSMUSG00000001158	*Snrnp27*	0.754	5.24E-05
	ENSMUSG00000071337	*Tia1*	0.854	2.16E-04
	ENSMUSG00000099354	*1700124L16Rik*	0.886	1.44E-03
	ENSMUSG00000004347	*Pde1c*	-0.512	5.67E-03
	ENSMUSG00000030091	*Nup210*	-0.668	7.21E-03
	ENSMUSG00000033860	*Fgg*	-1.206	7.34E-03
	ENSMUSG00000072849	*Serpina1e*	-1.217	7.34E-03
	ENSMUSG00000100226	*Gm20904*	1.321	8.71E-03
	ENSMUSG00000068299	*Nat8f4*	-0.555	1.27E-02
	ENSMUSG00000030895	*Hpx*	-1.330	2.03E-02
	ENSMUSG00000094230	*Gm21847*	1.335	2.03E-02
	ENSMUSG00000071178	*Serpina1b*	-1.051	2.24E-02
	ENSMUSG00000089871	*Speer4cos*	1.254	4.12E-02
	ENSMUSG00000006269	*Atp6v1b1*	-0.768	4.66E-02
C$^{+/+}$/*Tia1*$^{-/-}$ vs. *C*$^{+/+}$	ENSMUSG00000006269	*Atp6v1b1*	-1.134	1.50E-03
	ENSMUSG00000030314	*Atg7*	-0.696	1.76E-04
	ENSMUSG00000037541	*Shank2*	-0.637	3.48E-01
	ENSMUSG00000037855	*Zfp365*	1.166	4.30E-01
	ENSMUSG00000059430	*Actg2*	-0.775	3.78E-02
	ENSMUSG00000068744	*Psrc1*	1.320	4.55E-02
	ENSMUSG00000071337	*Tia1*	1.036	1.76E-04
	ENSMUSG00000074968	*Ano3*	1.390	1.14E-02
	ENSMUSG00000090141	*Gm614*	-0.950	4.30E-01

Supplementary Table 6: Top 50 upregulated genes in $C^{+/+}$ testis

**Accession**	**Gene Symbol**	**L2FC**	**Adjusted *p* value**
ENSMUSG00000023224	*Serping1*	1.807	1.03E-21
ENSMUSG00000020427	*Igfbp3*	2.204	5.07E-20
ENSMUSG00000003534	*Ddr1*	1.605	1.67E-18
ENSMUSG00000017466	*Timp2*	1.087	5.10E-17
ENSMUSG00000019539	*Rcn3*	1.809	6.05E-17
ENSMUSG00000025784	*Clec3b*	1.871	1.67E-16
ENSMUSG00000052911	*Lamb2*	1.549	3.61E-16
ENSMUSG00000036181	*Hist1h1c*	1.823	3.77E-16
ENSMUSG00000041801	*Phlda3*	2.126	2.24E-15
ENSMUSG00000021185	*9030617O03Rik*	2.270	2.43E-15
ENSMUSG00000055172	*C1ra*	1.828	2.48E-15
ENSMUSG00000029309	*Sparcl1*	1.570	4.40E-15
ENSMUSG00000020473	*Aebp1*	1.659	5.28E-15
ENSMUSG00000024186	*Rgs11*	1.920	5.28E-15
ENSMUSG00000033227	*Wnt6*	1.454	1.53E-14
ENSMUSG00000002985	*Apoe*	1.729	1.81E-14
ENSMUSG00000041117	*Ccdc8*	1.372	3.16E-14
ENSMUSG00000043719	*Col6a6*	2.040	5.46E-14
ENSMUSG00000024909	*Efemp2*	1.562	5.81E-14
ENSMUSG00000000753	*Serpinf1*	1.510	1.42E-13
ENSMUSG00000026170	*Cyp27a1*	1.461	1.75E-13
ENSMUSG00000002900	*Lamb1*	1.342	2.16E-13
ENSMUSG00000026836	*Acvr1*	1.553	2.24E-13
ENSMUSG00000019929	*Dcn*	1.693	2.71E-13
ENSMUSG00000021190	*Lgmn*	1.069	2.99E-13
ENSMUSG00000021876	*Rnase4*	1.504	9.27E-13
ENSMUSG00000078234	*Klhdc7a*	1.635	9.27E-13
ENSMUSG00000046805	*Mpeg1*	1.423	1.29E-12
ENSMUSG00000040612	*Ildr2*	2.117	2.20E-12
ENSMUSG00000053279	*Aldh1a1*	1.367	2.20E-12
ENSMUSG00000021091	*Serpina3n*	2.347	2.90E-12
ENSMUSG00000025223	*Ldb1*	1.200	3.77E-12
ENSMUSG00000017493	*Igfbp4*	1.338	4.52E-12
ENSMUSG00000025225	*Nfkb2*	1.539	4.52E-12
ENSMUSG00000004558	*Ndrg2*	1.330	6.11E-12
ENSMUSG00000031875	*Cmtm3*	1.275	6.57E-12
ENSMUSG00000020467	*Efemp1*	1.611	7.11E-12
ENSMUSG00000032531	*Amotl2*	1.318	8.54E-12
ENSMUSG00000006344	*Ggt5*	1.834	9.83E-12
ENSMUSG00000037852	*Cpe*	1.295	1.00E-11
ENSMUSG00000025511	*Tspan4*	1.538	1.03E-11
ENSMUSG00000030849	*Fgfr2*	1.552	1.15E-11
ENSMUSG00000026728	*Vim*	1.038	1.19E-11
ENSMUSG00000040740	*Slc25a34*	1.570	1.40E-11
ENSMUSG00000021268	*Meg3*	1.532	1.92E-11

Accession	Gene Symbol	L2FC	Adjusted *p* value
ENSMUSG00000053113	*Socs3*	2.113	1.92E-11
ENSMUSG00000033491	*Prss35*	1.712	2.26E-11
ENSMUSG00000014599	*Csf1*	1.509	2.97E-11
ENSMUSG00000055254	*Ntrk2*	1.540	3.01E-11
ENSMUSG00000029622	*Arpc1b*	1.151	3.14E-11

Supplementary Table 7: Top 50 downregulated genes in $C^{+/+}$ testis

**Accession**	**Gene Symbol**	**L2FC**	**Adjusted *p* value**
ENSMUSG00000008730	*Hipk1*	-0.553	3.96E-10
ENSMUSG00000042688	*Mapk6*	-0.546	9.84E-08
ENSMUSG00000040852	*Plekhh2*	-0.611	1.99E-07
ENSMUSG00000028519	*Dab1*	-1.497	1.68E-06
ENSMUSG00000042772	*Smg7*	-0.447	2.43E-06
ENSMUSG00000042350	*Arel1*	-0.503	1.02E-05
ENSMUSG00000026594	*Ralgps2*	-0.418	1.30E-05
ENSMUSG00000060726	*Tmsb15a*	-1.078	1.49E-05
ENSMUSG00000030510	*Cers3*	-0.430	3.03E-05
ENSMUSG00000028132	*Tmem56*	-0.958	3.68E-05
ENSMUSG00000047046	*1700031F10Rik*	-1.126	4.06E-05
ENSMUSG00000022155	*Mroh2b*	-0.446	4.98E-05
ENSMUSG00000034848	*Ttc21b*	-0.344	5.94E-05
ENSMUSG00000030994	*D7Ertd443e*	-0.613	6.13E-05
ENSMUSG00000048039	*Isg20l2*	-0.610	6.14E-05
ENSMUSG00000052221	*Ppp1r36*	-0.594	8.43E-05
ENSMUSG00000033767	*D930015E06Rik*	-0.622	9.00E-05
ENSMUSG00000039099	*Wdr93*	-0.391	9.51E-05
ENSMUSG00000008813	*Tppp2*	-0.922	1.00E-04
ENSMUSG00000062604	*Srpk2*	-0.527	1.02E-04
ENSMUSG00000020948	*Klhl28*	-0.585	1.02E-04
ENSMUSG00000024500	*Ppp2r2b*	-0.850	1.10E-04
ENSMUSG00000059114	*Lrrc74a*	-0.713	1.22E-04
ENSMUSG00000094556	*Gm20821*	-1.120	1.22E-04
ENSMUSG00000074746	*Pdzd8*	-0.692	1.25E-04
ENSMUSG00000039209	*Rpl39l*	-0.650	1.35E-04
ENSMUSG00000073730	*4933415F23Rik*	-0.601	1.43E-04
ENSMUSG00000029679	*Hyal6*	-0.778	1.56E-04
ENSMUSG00000101801	*1700020G17Rik*	-0.802	1.57E-04
ENSMUSG00000028706	*Nsun4*	-0.750	1.57E-04
ENSMUSG00000028287	*1700009N14Rik*	-0.778	1.62E-04
ENSMUSG00000021258	*Ccnk*	-0.438	1.62E-04
ENSMUSG00000071359	*Tbpl1*	-0.491	1.64E-04
ENSMUSG00000006527	*Sfmbt1*	-0.407	1.77E-04
ENSMUSG00000027227	*Sord*	-0.566	1.79E-04
ENSMUSG00000019945	*1700040L02Rik*	-0.459	1.93E-04
ENSMUSG00000021693	*Kif2a*	-0.537	1.95E-04
ENSMUSG00000033213	*AA467197*	-0.665	1.95E-04
ENSMUSG00000040456	*Hypm*	-0.916	1.98E-04
ENSMUSG00000027880	*Slc25a54*	-0.570	2.05E-04
ENSMUSG00000004798	*Ulk2*	-0.390	2.12E-04
ENSMUSG00000072295	*Als2cr11*	-0.558	2.22E-04
ENSMUSG00000025519	*Tktl2*	-0.478	2.34E-04
ENSMUSG00000026575	*Nme7*	-0.520	2.40E-04
ENSMUSG00000039000	*Ube3c*	-0.391	2.40E-04

Accession	Gene Symbol	L2FC	Adjusted *p* value
ENSMUSG00000031536	*Polb*	-0.560	2.48E-04
ENSMUSG00000029155	*Spata18*	-0.783	2.51E-04
ENSMUSG00000052371	*Hoxd3os1*	-0.537	2.52E-04
ENSMUSG00000029279	*Brdt*	-0.395	2.53E-04
ENSMUSG00000031864	*Ints10*	-0.453	2.55E-04

Supplementary Table 8: Top 50 upregulated genes in $C^{+/+}/Tia1^{-/-}$ testis

**Accession**	**Gene Symbol**	**L2FC**	**Adjusted *p* value**
ENSMUSG00000023224	*Serping1*	2.060	3.25E-41
ENSMUSG00000021185	*9030617O03Rik*	3.028	6.86E-35
ENSMUSG00000017466	*Timp2*	1.197	2.19E-31
ENSMUSG00000020427	*Igfbp3*	2.372	4.34E-31
ENSMUSG00000023067	*Cdkn1a*	1.936	8.29E-31
ENSMUSG00000026170	*Cyp27a1*	1.841	2.76E-30
ENSMUSG00000020467	*Efemp1*	2.146	9.20E-29
ENSMUSG00000003534	*Ddr1*	1.614	3.61E-27
ENSMUSG00000029309	*Sparcl1*	1.743	1.07E-26
ENSMUSG00000019929	*Dcn*	2.005	6.10E-26
ENSMUSG00000002985	*Apoe*	1.928	2.91E-25
ENSMUSG00000041801	*Phlda3*	2.382	3.04E-25
ENSMUSG00000024186	*Rgs11*	2.137	6.36E-25
ENSMUSG00000036181	*Hist1h1c*	1.916	6.81E-25
ENSMUSG00000053279	*Aldh1a1*	1.612	6.81E-25
ENSMUSG00000025784	*Clec3b*	1.934	3.43E-24
ENSMUSG00000019539	*Rcn3*	1.800	2.28E-23
ENSMUSG00000055172	*C1ra*	1.901	8.20E-23
ENSMUSG00000048583	*Igf2*	1.842	1.02E-22
ENSMUSG00000052911	*Lamb2*	1.498	4.99E-22
ENSMUSG00000000753	*Serpinf1*	1.625	8.57E-22
ENSMUSG00000021876	*Rnase4*	1.662	1.07E-21
ENSMUSG00000002900	*Lamb1*	1.413	1.99E-21
ENSMUSG00000032531	*Amotl2*	1.492	2.75E-21
ENSMUSG00000006800	*Sulf2*	1.416	3.04E-21
ENSMUSG00000032609	*Klhdc8b*	1.703	8.90E-21
ENSMUSG00000078234	*Klhdc7a*	1.796	1.11E-20
ENSMUSG00000043719	*Col6a6*	2.178	1.22E-20
ENSMUSG00000032788	*Pdxk*	1.495	1.39E-20
ENSMUSG00000033227	*Wnt6*	1.444	1.73E-20
ENSMUSG00000046805	*Mpeg1*	1.511	2.60E-20
ENSMUSG00000001986	*Gria3*	2.260	5.09E-20
ENSMUSG00000021190	*Lgmn*	1.080	6.28E-20
ENSMUSG00000017493	*Igfbp4*	1.432	8.86E-20
ENSMUSG00000022894	*Adamts5*	2.020	1.56E-19
ENSMUSG00000022330	*Osr2*	1.871	1.61E-19
ENSMUSG00000030849	*Fgfr2*	1.699	3.96E-19
ENSMUSG00000004558	*Ndrg2*	1.409	4.37E-19
ENSMUSG00000031875	*Cmtm3*	1.363	5.26E-19
ENSMUSG00000001750	*Tcirg1*	1.769	5.37E-19
ENSMUSG00000028517	*Plpp3*	1.382	8.73E-19
ENSMUSG00000006344	*Ggt5*	2.031	9.01E-19
ENSMUSG00000037852	*Cpe*	1.357	1.15E-18
ENSMUSG00000032000	*Birc3*	1.635	2.00E-18
ENSMUSG00000040564	*Apoc1*	2.044	2.06E-18

**Accession**	**Gene Symbol**	**L2FC**	**Adjusted *p* value**
ENSMUSG00000014599	*Csf1*	1.642	2.18E-18
ENSMUSG00000033491	*Prss35*	1.865	2.81E-18
ENSMUSG00000068744	*Psrc1*	2.765	2.94E-18
ENSMUSG00000041117	*Ccdc8*	1.307	3.05E-18
ENSMUSG00000026836	*Acvr1*	1.543	4.17E-18

Supplementary Table 9: Top 50 downregulated genes in $C^{+/+}/Tia1^{-/-}$ testis

**Accession**	**Gene Symbol**	**L2FC**	**Adjusted *p* value**
ENSMUSG00000008730	*Hipk1*	-0.676	7.18E-23
ENSMUSG00000040852	*Plekhh2*	-0.812	3.31E-19
ENSMUSG00000030314	*Atg7*	-0.915	2.37E-17
ENSMUSG00000042688	*Mapk6*	-0.632	1.58E-15
ENSMUSG00000057286	*St6galnac2*	-1.141	3.31E-14
ENSMUSG00000059430	*Actg2*	-1.187	3.96E-14
ENSMUSG00000029155	*Spata18*	-1.224	5.75E-14
ENSMUSG00000042350	*Arel1*	-0.648	7.81E-14
ENSMUSG00000033767	*D930015E06Rik*	-0.895	1.13E-13
ENSMUSG00000048039	*Isg20l2*	-0.854	1.60E-13
ENSMUSG00000028287	*1700009N14Rik*	-1.163	1.67E-13
ENSMUSG00000070639	*Lrrc8b*	-1.051	3.60E-13
ENSMUSG00000074746	*Pdzd8*	-0.988	6.71E-13
ENSMUSG00000029766	*1700012A03Rik*	-1.378	8.84E-13
ENSMUSG00000008813	*Tppp2*	-1.317	1.19E-12
ENSMUSG00000024500	*Ppp2r2b*	-1.209	1.30E-12
ENSMUSG00000018796	*Acsl1*	-1.076	1.85E-12
ENSMUSG00000028141	*Oaz3*	-1.248	2.02E-12
ENSMUSG00000028706	*Nsun4*	-1.064	2.48E-12
ENSMUSG00000031545	*Gpat4*	-1.002	3.18E-12
ENSMUSG00000027227	*Sord*	-0.789	4.57E-12
ENSMUSG00000066368	*Actl11*	-0.975	5.05E-12
ENSMUSG00000074435	*Smcp*	-1.217	5.05E-12
ENSMUSG00000017119	*Nbr1*	-0.921	5.98E-12
ENSMUSG00000051437	*Ubqlnl*	-1.068	8.30E-12
ENSMUSG00000010461	*Eya4*	-0.977	1.01E-11
ENSMUSG00000061923	*Odf1*	-1.208	1.01E-11
ENSMUSG00000031085	*Gm498*	-1.153	1.27E-11
ENSMUSG00000021097	*Clmn*	-1.066	1.36E-11
ENSMUSG00000030994	*D7Ertd443e*	-0.788	1.64E-11
ENSMUSG00000059114	*Lrrc74a*	-0.957	1.69E-11
ENSMUSG00000090457	*4930571K23Rik*	-1.100	1.97E-11
ENSMUSG00000028132	*Tmem56*	-1.219	2.90E-11
ENSMUSG00000029320	*1700016H13Rik*	-1.070	3.81E-11
ENSMUSG00000049008	*BB014433*	-1.037	3.84E-11
ENSMUSG00000006269	*Atp6v1b1*	-1.358	4.58E-11
ENSMUSG00000036918	*Ttc7*	-1.088	8.12E-11
ENSMUSG00000038587	*Akap12*	-0.786	9.88E-11
ENSMUSG00000038057	*Dbil5*	-1.106	1.22E-10
ENSMUSG00000027286	*Lrrc57*	-1.073	1.26E-10
ENSMUSG00000031770	*Herpud1*	-0.886	1.58E-10
ENSMUSG00000001052	*Sec24b*	-0.615	1.64E-10
ENSMUSG00000071644	*Eef1g*	-0.729	1.78E-10
ENSMUSG00000004798	*Ulk2*	-0.503	1.90E-10
ENSMUSG00000026778	*Prkcq*	-0.853	2.05E-10

**Accession**	**Gene Symbol**	**L2FC**	**Adjusted *p* value**
ENSMUSG00000050089	*Akap4*	-1.004	2.10E-10
ENSMUSG00000100315	*1700031P21Rik*	-1.057	2.36E-10
ENSMUSG00000039033	*Tasp1*	-0.728	2.36E-10
ENSMUSG00000031647	*Mfap3l*	-1.018	3.61E-10
ENSMUSG00000054909	*Wbscr25*	-1.064	3.63E-10

Supplementary Table 10: QPCR Primer List

Gene	Forward Primer	Reverse Primer
1700124L16Rik	GATGGTCAGCTACGGGGAAG	AGGGGTGGGAGCTTTCAAAC
Acin1	AAGAAACCGTCCATCAGTATC	CCATTACGCTCTGTCTCATC
Actg2	CGTACCACAGGCATCGTTCT	CAAGACGCATGATGGCATGG
Aifm1	CCGTTCGGAGAGTGAGACAGAG	TAGCACAATCCCCACCACAACT
Akt1	ACACCTTTATCATCCGCTGCCT	CTCTTCAGCCCCTGAGTTGTCA
Angptl4	ATTGTTCCAGAAGGTGGCCC	GCCAAGAGGTCTATCTGGCTC
Ano3	TCCTTTAAACAGCAAAAAGGTATGA	AGGCAGGGATCTCCTAGATGG
Apaf1	GAGATCCACACAGGCCATCAC	ATCACACCGTGAACCCAACTCA
Apoe	GTCACATTGCTGACAGGATGCC	CTTCCTGGACCTGGTCAGACAG
Atg7	AGCGGCGACAGCATTAGGAT	CCTCATGGCAGGAAAGCAGT
Atp6v1b1	TCAGAAGACATGCTGGGTCG	TCTCCTCGGGGTAGATTCGG
Bax	CCCACCAGCTCTGAACAGATCA	TGTCCACGTCAGCAATCATCCT
Bcl3	ACAGCGGCCTCAAGAACTGT	TGGCACTTTGGTCTGGGGAT
Capn2	TTGACGCCAATGAGGAGGACAT	CCACTCCCATCTTCATCCAGCA
Casp7	AACCGTCCACAATGACTGCTCT	TGTCACGCCATCTTTCCCGTAA
Casp8	AGCTGCGGGATCCAGACAAT	TGCCAGCATGGTCCTCTTCT
Casp9	GGACCGTGACAAACTTGAGCAC	ATCTCCATCAAAGCCGTGACCA
Cntn4	CTGGAAGTTACACCTGCATAG	GAGGAACCATCACCTTTGTT
Cpe	TCTCTGTGGACGGGATAGACCA	CAAGCTCAAAGTCCACCCCAAC
Cxcl12	GGTAAACCAGTCAGCCTGAGCT	TTGTTGTTCTTCAGCCGTGCAA
Ddr1	GACTCCTCTGACACCTTCCCAC	TGAGAAGCAGCAGCAGGATGAT
Dhcr24	GATGGACATCCTGGAGGTGGAC	GGTCATCAAGCTCAGGCAACAC
Dhcr7	GCCTGGACCCTCATTAACCTGT	AAGTGGTCATGGCAGATGTCGA
Efnb1	AACGTCCAATGGGAGCTTGGAG	TAGTCAACTGCTCGGGTGTCAC
Ephb1	GCAGCAGGAAACGAGCTTACAG	CGGCTTCATTGGGGTCCTCATA
Fgg	GACGGCATTATTTGGGCGAC	AACGTCTCCAGCCTGTTTGG
Gapdh	TGCCCCCATGTTTGTGATG	TGTGGTCATGAGCCCTTCC
Gm15631	GGAGATGGAAGTGGCTTATTT	GTTCTCTGAGCAAGGAACTG
Gm20904	TGCCATTCTTACAGGACTATTT	GGTTACCTTCCTTGTAGTCATC
Gm21847	CGGCTGTGCAGGTTACTGT	GGCATTTTTCCTTCTCTGTGGT
Gm614	TTCATGCCCAACCCCTATGC	ACTCTTGATTGTCTCCATGTTTGA
Gm9999	GGGACCCCTCTGGAAGTAAACA	TGTGCGATTACTTTATGCGAAACCT
Herc6	ACCGAAAGGCCAGAACCAAT	CTCCCCAAGCGAAGACCTTT
Hist1h2bc	GATCCTGCCAAGAGGAGCAG	CCCATTTGCACTGTCTTGAGG
Hist1h4h	CCTGTGCCTTCCACTCTGTT	CCAAGACCTTTACCGCCCTT
Hpx	CCTCTTCCTACTGCCAACGG	TCCATGGTCGCAGCATCAAA
Itgb1	GTGAGACATGTCAGACCTGCCT	TCCTTGCAATGGGTCACAGGAT
Lamb2	GGGTGAGAGACAGAAGGCAGAG	ACCGGCAGAGTTCAGAGACTTC
Lancl2	CTGCATATACTGGTTGGACAG	GGAGTGTTCTCTTCACGTAATC
Lipa	AATTTTGCTTCAGGCCCGCTAC	GCGCAAAGCTCCTTCATGATGA
Lss	AGGAGCACGTTTCTCGGATCAA	AGGGCAGAAACTCAGGTCTGTG
Malat1	TTCATGGAGCTGCTCAGGACTT	ACCTGAAGTCAAGACACCTGCA
Meg3	CCATCTCCACAGAAGAGCAGCT	CCCACGCAGGATTCCAGATGAT
Mfsd2a	TGGATGTGGCTAAGGTGGAAC	GAGGGTCAGTGAAGGCATCC
Msmo1	GCTGTGCAGTCATTGAGGACAC	GGGATGTGCGTATTCTGCTTCG
Nagk	ATGCCCGGTGCCTTTGTG	CCAATCAGCCAGTGGTTTGTG
Nat8f4	GAAAAGTGTCCGCAAGGCAG	CTGACTACTTGGGCGGGAAC
Nat8f5	CCAGACCCTGAAGAAGAAAC	CTTGAGTCCTCCAAGGAATTG
Neat1	TGTTCACCATGGCAAGCAGATG	ACAAGCTGACTTCAACGATGGC
Ntn1	TGTCTCAACTGCCGCCACAA	TTGCAGGGACATTGGCCAGT

Gene	Forward Primer	Reverse Primer
Ntn3	ATGCCGCTTCAACATGGAGC	TGGCAGTCACAAGCTCTGCA
Nup210	AGCACAGCCCCCACTATCTT	TGCACATTGCCTAGTGAGAGG
Oaz3	GGATCAAGGCAACCGAGAAAGC	TCTAACCACCTCGAAGCCCATG
Parva	GACGTATTGGTGGGAGAAAG	GAAACTGGAGAGTGAGAAGC
Pcyox1	GGTTTCAGTCCCTCAGAATG	TTCTACACTGCTAAAGGCATAG
Pde1c	AGTGTTATCCCCTCCCTCCC	AAGAGGAGAACAGCAAGCCC
Pisd-ps1	CAAAGCCCACAGGCTAAA	ATCCCTCCTGCACTACAA
Plin4	AGGCAAGACCTTGAGCAGTTT	AGGGGTCTGTTGCTGTTTGT
Pnpla2	GAGGAATGGCCTACTGAACC	AGGCTGCAATTGATCCTCCT
Pnpla7	AGCACGTATTCCAACCTGGG	TGCCATCAGCGTCTTGGATG
Ppp2r2b	ACCGGAAGATCCAAGCAACAGA	AACTTGCTGCGGAGGTAGTCAT
Ppp3r2	GCTGGTGGACAAGAGCATCTT	AAGTCTTCTTGACCGTGTTCTACA
Prrxl1	GTCTTCACCAGAGAAGAGTTAG	CTCTCTGTCTTCCTCCATTTG
Psrc1	AGACTGTGAAGCGAGGTCTAAG	GAAGTCCAAGGTCTCGTCCA
Pyurf	CCGCTTAGATATGAAGCATCG	CTCAGCTTCTTCTTGCTTCTC
Rims4	GAGATTGGCTTGCAGGAG	CAGCAGATAGGCCTTGATATAG
Robo1	CTCCCCCACATAGCAACAGTGA	GTAGGACACAGCAGCTGGAGAA
RP23-402F16	ATGAAGACTTCTCCACCCAGCC	CCACCCTACCTACTGTAGTACGC
Rph3a	GCCAGCAAGTCCAACAA	GCATGTCCTCCTCTGTAATG
Rxrg	CCAGATGCCAAGGGTTTAT	CGGATACTTCTGCTTGGTATAG
Sema7a	CGGAAGCTCTATGTGACCTCCC	GTGTGGCTCCGCTGGATTAATG
Serpina1b	AGCTCAGCAAGGCTGTGCATAA	GCATAGACATAGGAACGGCTTCA
Serpina1e	AAGAGGCCAAGAACCATTATC	TTCCTTGGGTTCCCTTCT
Serping1	CCACTTACCTGACGATGCCTCA	TGGCAGACACCTGAAGATCTGG
Shank2	CGCTATCCCCGGAATTCTCTC	GGTATCGGCTTTTGCCCCT
Six3	CAGCAAGAAACGCGAACT	TGCTGGAGCCTGTTCTT
Slit3	GTGGCGACTGACAAGGACAATG	CCACACTGTGAAACTGGCCATC
Snrnp27	AAAGAAGACGCCGAGAAAGGT	TATGTCGTCTTGGAGACCTGGA
Speer4cos	ACACTCTGCATGGGGATGGA	GTTGAGCTTCCTGGTAGGGG
Spr-ps1	TCCAGACAAAGAGTGTGGCA	CGTGCGTTTAGGAGCATCAC
Srgap1	CTGTACTTCCGAGGGCTGGAAA	CGTCACTGTACTGGGAGAGGTG
Tbc1d1	AGCACAGCCAGCTGAGGA	GAGGGCAGAATCCAGTGAGG
Tbc1d24	CCATCCGCCTATTTTCCCGA	AGTGCACAAACTGCCTTTTAGA
Tppp2	GGCCAAGAATGCCAGAACCATC	TGGTGTCTGTCAACCGGTCTAC
Unc5b	ACGGCCAACTACACCTGTGT	ACAGAAGGCGCCTCCATTGA
Zfp365	TCAAGGAAAAGCGAGGCTCC	CGATGGGATGTCTGTGAGCATA

Supplementary Materials and Methods

Protein isolation and immunoblotting

P42 brain was homogenized in ten volumes of radio immunoprecipitation (RIPA) buffer (Boston Biotechnologies) with 1X HALT protease inhibitor (Thermo Fisher Scientific) using a Microson Ultrasonic Cell Disrupter set at speed 2 (Misonix). The homogenate was incubated on ice for 30 minutes and then centrifuged at 12,000 x g for 15 minutes at 4°C and the supernatant transferred to a new tube. Protein concentration was determined with the Protein Dye Assay Concentrate (BioRad). For P42 spinal cord, heart, and uterus/ovaries, samples were initially briefly homogenized in 10 volumes RIPA buffer with HALT protease inhibitor. Two hundred μL of the homogenate was transferred to 500 μL Trizol and briefly homogenized. This homogenate was used to obtain RNA from the tissue following the procedure above. The remaining RIPA homogenate was subsequently sonicated twice more as above and protein supernatant obtained and concentration determined as described above.

Protein lysates were diluted with 2X Laemlli sample buffer (BioRad) with 10% β-mercaptoethanol (Sigma-Aldrich). Samples were boiled for five minutes and then centrifuged at maximum speed for five minutes at room temperature. Thirty μg protein was separated on 10% SDS-PAGE gels. The separated protein was transferred to Immobilon polyvinylidene fluoride (PVDF) membrane (Millipore) using the Trans-Blot Turbo system (BioRad). Membranes were incubated in 5% nonfat milk (Walmart) prepared in 1X Tris-buffered saline with Tween 20 (TBST; 50 mM Tris; 150 mM NaCl, 0.05% Tween-20) and subsequently incubated in primary antibody. Primary antibodies were: mouse anti-SMN (BD Biosciences; 610646) diluted 1:1000; donkey anti-TIA1 (Santa Cruz Biotechnologies; sc-1751) diluted 1:250; donkey anti-TIAR (Santa Cruz Biotechnolgies; sc-1749) diluted 1:250; mouse anti-Gemin2 clone 2E17 (Sigma;

G6669) diluted 1:750; rabbit anti-β-actin (Sigma; A2103) diluted 1:200. Primary incubations were performed either overnight at 4°C with shaking (SMN, TIA1, TIAR, Gemin2) or for 1 hour at room temperature (β-actin). The blots were then washed with TBST and incubated in secondary antibody diluted 1:4000 in 5% milk for 1 hour at room temperature. Secondary antibodies were: goat anti-mouse IgG (Jackson Immunoresearch; 115-035-003); donkey anti-goat IgG (Santa Cruz Biotechnologies; sc-2020) or donkey ant-rabbit IgG (GE Healhcare; NA934V) all conjugated to horseradish peroxidase (HRP). Protein was detected using WestFemto substrate (Thermo Fisher Scientific) and visualized with the BioSpectrum AC Imaging System (UVP). For each blot, the mean intensity of each band was determined using ImageJ software. The SMN, Tia1, Tiar or Gemin2 mean intensities were then divided by the β-actin mean intensity for the same lane to normalize the data and expressed as a percent of WT.
